# A novel aquaporin Aagp contributes to *Streptococcus suis* H_2_O_2_ efflux and virulence

**DOI:** 10.1080/21505594.2023.2249789

**Published:** 2023-08-24

**Authors:** Xinchi Zhu, Shuoyue Wang, Yu Du, Zijing Liang, Huochun Yao, Xiang Chen, Zongfu Wu

**Affiliations:** a MOE Joint International Research Laboratory of Animal Health and Food Safety, College of Veterinary Medicine, Nanjing Agricultural University, Nanjing, China; b Key Lab of Animal Bacteriology, Ministry of Agriculture, Nanjing, China; c OIE Reference Lab for Swine Streptococcosis, Nanjing, China; d Jiangsu Key Laboratory of Zoonosis, Yangzhou University, Yangzhou, Jiangsu, China

**Keywords:** *Streptococcus suis*, oxidative stress, aquaporin, virulence, glycerol transport

## Abstract

*Streptococcus suis* is a bacterium that can cause infections in pigs and humans. Although oxidative stress is common occurrence during bacterial growth and infection, the regulation networks of *S. suis* under oxidative stress remain poorly understood. To address this, we utilized RNA-Seq to reveal the transcriptional landscape of *S. suis* in response to H_2_O_2_ stress. We identified novel genes responsible for *S. suis* resistance to oxidative stress, including those involved in DNA repair or protection, and essential for the biosynthesis of amino acids and nucleic acids. In addition, we found that a novel aquaporin, Aagp, belonging to atypical aquaglyceroporins and widely distributed in diverse *S. suis* serotypes, plays a crucial role during H_2_O_2_ stress. By performing oxidative stress assays and measuring the intracellular H_2_O_2_ concentrations of the wild-type strain and *Aagp* mutants during H_2_O_2_ stress, we found that Aagp facilitated H_2_O_2_ efflux. Additionally, we found that Aagp might be involved in glycerol transport, as shown by the growth inhibition and H_2_O_2_ production in the presence of glycerol. Mice infection experiments indicated that Aagp contributed to *S. suis* virulence. This study contributes to understanding the mechanism of *S. suis* oxidative stress response, *S. suis* pathogenesis, and the function of aquaporins in prokaryotes.

## Introduction

*Streptococcus suis* can cause systemic diseases such as septicaemia and meningitis in pigs. It is also considered as a zoonotic pathogen for humans in close contact with infected pigs or contaminated by-products [1–3]. During the growth and infection process, *S. suis* may encounter oxidative stress. Reactive oxygen species (ROS) like hydroxyl radicals (HO•), hydrogen peroxide (H_2_O_2_), and superoxide anion (O_2_^−^) are generated during many cellular activities [4]. O_2_^−^ and H_2_O_2_ result from one-electron and two-electron reduction of O_2_, respectively [5,6]. H_2_O_2_ can oxidize Fe^2+^ in ferritin, leading to inactivation of the enzymes and releasing Fe^3+^, and then Fe^3+^ is reduced to Fe^2+^ in the intracellular environment [5]. When Fe^2+^ reacts with H_2_O_2_, Fenton chemistry is occurred and generates highly-reactive HO• [7]. O_2_^−^, H_2_O_2_, and HO• can cause cell damage by oxidizing amino acids, DNA, and lipids [5,8]. When bacteria invade the host, innate immune cells, including neutrophils, monocytes, and macrophages, are responsible for ROS production, which is essential to eliminate bacteria [9]. In addition, ROS participates as a signal molecular in activating various immune mechanisms [10]. In order to defend against oxidative stress, *S. suis* has developed various mechanisms that are regulated by transcriptional regulators, including PerR, Rex, SpxA1, FlpS, and SrtR, which target genes encoding superoxide dismutase, catalase, thioredoxin, glutathione reductase, iron uptake (Fur), and ferritin [11–15]. However, since antioxidant defence systems involve comprehensive and complex processes, studies focusing solely on individual regulators cannot fully reveal regulation networks under oxidative stress.

In this study, we performed RNA-sequencing (RNA-Seq) analysis to explore the global oxidative stress response of *S. suis*. Our findings revealed a comprehensive defence network in response to H_2_O_2_ stress, and we identified a novel aquaporin, Aagp, that plays a crucial role in facilitating *S. suis* H_2_O_2_ efflux and contributes to virulence.

## Materials and methods

### Bacterial strains and culture conditions

Supplementary Table S1 lists *S. suis* strains and plasmids. *S. suis* serotype 9 virulent strain GZ0565 was isolated from a meningitis pig [16]. *S. suis* strains were cultured in Todd-Hewitt broth (THB, Hopebio, Qingdao, China) and plated on an agar (THA) medium containing 6% (vol/vol) sheep blood at 37 °C with 5% CO_2_. *Escherichia coli* strains were grown in Luria-Bertani (LB, Becton Dickinson, USA) broth at 37 °C. Antibiotics were added when in need as follows: spectinomycin (Spc, Macklin, China), 50 μg/mL for *E. coli*, and 100 μg/mL for *S. suis*, 10% (w/v) sucrose (sucrose, Macklin, China) for *S. suis*.

For studying glycerol utilization, *S. suis* strains were grown in THB to mid-exponential phase (OD_600_ = 0.6), washed twice in PBS, and then transferred (with a dilution of 1:100) to a 50 mL flask with 10 mL of 1/3 diluted THB or 1/3 diluted THB containing glycerol with a final concentration of 10 mM or 100 mM. The growth rates were measured at OD_600_ at 37 °C in a shaking incubator.

### RNA-Seq analysis

To serve as a control group, *S. suis* strain GZ0565 was grown in THB to reach the mid-exponential phase. For H_2_O_2_ treatment group, when the bacteria had reached the mid-exponential phase, a final concentration of 25 mM H_2_O_2_ was added to THB medium and incubated for 25 min. After incubation, the cultures were centrifuged at 5000×*g* for 10 min at 4 °C, and the bacterial pellets were collected. The total RNA was extracted using the FastRNA Pro Blue Kit (MP Biomedicals) according to our previous report [17]. The extracted RNA samples were submitted to Realbio Technology (Shanghai, China) for RNA-seq analysis. Each experimental group was performed in duplicate. The library construction for RNA-seq was described in our previous study [18]. Sequencing was carried out using Illumina HiSeq 2500 (Illumina, USA) following the manufacturer’s protocol. The sequencing reads were aligned by Bowtie2 [19]. The gene expression was calculated by FPKM (Fragments Per Kilobase of transcript per Million mapped reads), and differential expression analysis was performed using edgeR [20]. The threshold of *p* < 0.01 and the absolute value of Fold change ≥ 2 were used to identify the differentially expressed genes.

### Construction mutant strains

The detailed protocol for deleting *Aagp* (*BFP66_RS01250*) in strain GZ0565 background using *S. suis-E. coli* shuttle plasmid pSET4s was described in our previous study [21]. In addition, we created a mutation strain of Aagp in strain GZ0565 background (Aagp^mut^) by inserting single “G” base into its coding sequence (CDS), causing a frameshift that rendered Aagp non-functional. This mutation strain was constructed through a two-step natural transformation [22]. Step I was performed to replace the upstream sequence of *Aagp* with *SacB-Spc* cassette and resulted in sucrose sensitive and spectinomycin resistant; step II was the process of cassette replacement for single base insertion via the negative selection on sucrose THA plate (Supplementary Figure S1). We also employed the same methodology to construct deletions of *exodeoxyribonuclease III* (*exo III, BFP66_RS05940*), *AguB* (*BFP66_RS06520*), *metQ* (*BFP66_RS08225*) in strain GZ0565 background, as well as *Aagp* in serotype 2 strain P1/7 background (*Aagp*_*P1/7*_). Supplementary Table S2 contains a list of primers used for constructing all these strains.

### Oxidative stress assays

To assess the role of the Aagp in oxidative stress response, *S. suis* strains were challenged with H_2_O_2_. Wild-type strain GZ0565 (WT), Δ*Aagp*, and Aagp^mut^ were cultured to the mid-exponential phase. Then the final concentration of 25 mM H_2_O_2_ was added to THB. After incubation at 37 °C for 25 min, the number of bacteria was determined by spreading serial dilutions on THA plates. The survival rate at each time point was calculated as CFU at time point 25 min/CFU at time point 0. The functions of *exo III*, *AguB*, *metQ* and *Aagp_P_*_*1*_*_/7_* in response to oxidative stress were evaluated using the same methodology. Experiments were performed with three biological replicates. The statistical analysis was performed with a two-tailed unpaired *t* test.

To further assess the susceptibility of WT, Δ*Aagp*, and Aagp^mut^ to H_2_O_2_, the Oxford cup supplemented with 10 µL 1 M H_2_O_2_ was conducted on the THA plates. After incubation at 37 °C with 5% CO_2_ for 24 h, a transparent circle formed, indicating an inhibition zone.

### Measurement of H_2_O_2_ in cells

After treated with 25 mM H_2_O_2_ for 25 min, one mL bacterial cultures were centrifuged at 5000 × *g* at 4 °C for 10 min and subsequently washed once with PBS and resuspended in 1 mL PBS. According to the manufacturer’s protocol, the concentration of H_2_O_2_ in cells was measured using the Pierce Quantitative Peroxide Assay Kits (Thermo Fisher Scientific, Shanghai, China). Experiments were performed with two biological replicates and repeated two times. A two-tailed unpaired *t* test was used for statistical analysis.

### Determination of viable bacteria in organs

The virulence of WT and Δ*Aagp* was assessed in mice according to our previous report [23]. Mice infection was carried out in the Laboratory Animal Center of Nanjing Agricultural University with the approval of the institution’s ethics committee (Permit number SYXK (Su) 2021–0086). Bacteria were cultured to the mid-exponential phase and washed twice with PBS. Six-week SPF CD1 female mice (SiPeiFu Biotechnology Co., Ltd, China) were used for infection (five mice per group). The mice were intraperitoneally injected with a dose of 1.5 × 10^8^ CFU of the WT or Δ*Aagp*. All mice were euthanized at 24 h post-infection. Blood samples were collected from the heart, and liver, kidney, and brain samples were taken, weighed, suspended in PBS, and homogenized. Plating serial dilutions on THA determined the number of viable bacteria in organs and blood. Experimental results were shown as mean ± standard error of the mean (SEM). The statistical analysis was performed with a two-tailed unpaired *t* test.

### RNA extraction and quantitative real-time PCR (RT-qPCR)

H_2_O_2_ treatment and RNA extraction were performed as described above for RNA-Seq analysis. The detailed protocol for RT-qPCR was described previously [18]. Supplementary Table 2 lists primers for RT-qPCR analysis. The gene *BFP66_RS5620* (*ParC*) was used as the internal control. Three biological replicates were used. The 2^−ΔΔCT^ method was used to calculate the relative fold change. Data were shown as mean ±SEM.

## Results

### *The transcriptional landscape of* S. sui*s oxidative response*

The RNA-Seq was used to explore the transcriptional landscape of *S. sui*s in response to H_2_O_2_. Compared with the control condition in the THB medium, 371 differently expressed genes (DEGs) were observed in the H_2_O_2_ treatment condition, consisting of 112 upregulated and 259 downregulated genes (Supplementary Table 3). The putative functions of these genes were classified into different functional categories according to the gene ontology (GO) (Supplementary Figure S2A). The DEGs are involved in various biological processes, such as biosynthesis of amid acids, carbohydrate transport and metabolism, metal ion transport, and transcriptional regulation.

**Figure 1.**
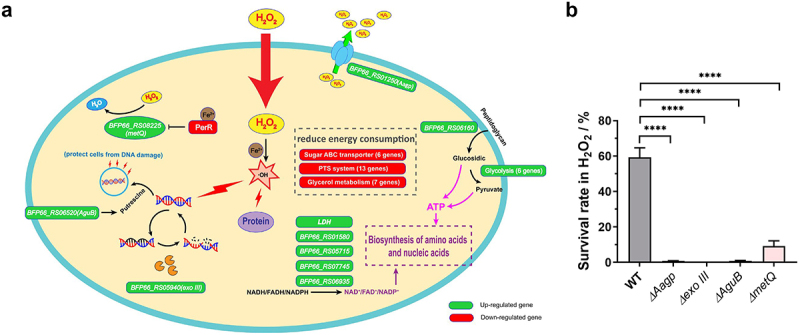
**The transcriptional landscape of *S. sui*s oxidative response**. (a) RNA-Seq analysis was performed to explore the global H_2_O_2_ stress response of *S. suis*. The upregulated genes are shown in green, and the downregulated genes are shown in red. The detailed information and fold change of genes shown in this figure are summarized in table 1. (b) The survival rate of WT, Δ*Aagp*, Δ*exo III*, Δ*AguB*, and Δ*metQ* was determined after 25 min treated with 25 mM H_2_O_2_. The statistical analyses were performed with a two-tailed unpaired *t* test. “****” indicate *p* < 0.001.

As shown in Figure 1a, we found that genes involved in DNA repair or protection were upregulated under H_2_O_2_ stress, including *BFP66_RS05940* encoding an exodeoxyribonuclease III (exo III) and *BFP66_RS06520* encoding an N-carbamoylputrescine amidase (AguB). The gene expression of enzymes encoded by *BFP66_RS01580, BFP66_RS05715, BFP66_RS06935, BFP66_RS07745, BFP66_RS04160* (*LDH*) was upregulated under H_2_O_2_ condition, and these enzymes contribute to producing oxidized dinucleotide cofactors (NAD^+^, FAD^+^, and NADP^+^) which are essential for the biosynthesis of amino acids and nucleic acids. As shown in Table 1 and Figure 1, six genes involved in glycolysis were upregulated under H_2_O_2_ condition, contributing to producing more ATP. In contrast, several genes related to energy-consuming pathways were downregulated, including six genes related to sugar ABC transporters, 13 related to the PTS system, and seven related to glycerol metabolism. RT-qPCR was performed to verify the transcriptional data. Eight upregulated and four downregulated genes were selected, and their expressions were consistent with those obtained from the RNA-seq (Supplementary Figure S2B).

**Table 1.** The key differentially expressed genes under H_2_O_2_ stress^a^.

ID	Fold Change (H_2_O_2_/THB)	Product	Function
*BFP66_RS01250*	3.66	aquaporin Aagp;	H_2_O_2_ efflux
*BFP66_RS01545*	−3.62	transcriptional regulator PerR;	oxidative stress regulation
*BFP66_RS09925*	−1.93	transcriptional regulator mntR;	oxidative stress regulation
*BFP66_RS08225*	1.35	methionine ABC transporter, MetQ	methionine transporter, converting H_2_O_2_ to H_2_O
*BFP66_RS06520*	2.01	N-carbamoylputrescine amidase;	converting agmatine to putrescine, which protects DNA from damage
*BFP66_RS05940*	2.89	exodeoxyribonuclease III;	DNA repair
*BFP66_RS06160*	2.19	N-acetyl-beta-hexosaminidase;	producing sugar by cell wall hydrolyzation
*BFP66_RS04160*	2.61	L-lactate dehydrogenase LDH;	generating NAD^+^/FAD^+^/NADP^+^ for biosynthesis of amino acids and nucleic acids
*BFP66_RS01580*	2.02	NADP-dependent oxidoreductase;	
*BFP66_RS05715*	2.30	NADH oxidase;	
*BFP66_RS07745*	2.09	nicotinate phosphoribosyltransferase;	
*BFP66_RS06935*	2.21	flavodoxin;	
*BFP66_RS01665*	3.07	fructose-bisphosphate aldolase;	glycolysis
*BFP66_RS07495*	2.61	2,3-bisphosphoglycerate-dependent phosphoglycerate mutase;	
*BFP66_RS06470*	2.19	pyruvate kinase;	
*BFP66_RS06865*	2.36	enolase;	
*BFP66_RS02590*	2.86	triose-phosphate isomerase;	
*BFP66_RS00790*	2.16	type I glyceraldehyde-3-phosphatedehydrogenase;	
*BFP66_RS00845*	−2.35	sugar ABC transporter substrate-binding protein;	sugar ABC transporters
*BFP66_RS00850*	−2.22	sugar ABC transporter permease;	
*BFP66_RS02995*	−8.65	sugar ABC transporter permease;	
*BFP66_RS07040*	−15.41	sugar ABC transporter permease;	
*BFP66_RS07045*	−10.83	sugar ABC transporter substrate-binding protein;	
*BFP66_RS08960*	−2.20	sugar ABC transporter substrate-binding protein;	
*BFP66_RS00910*	−7.00	PTS sugar transporter subunit IIC;	PTS system
*BFP66_RS02210*	−3.46	PTS system mannose/fructose/N-acetylgalactosamine-transporter subunit IIB;	
*BFP66_RS02215*	−1.90	PTS mannose/fructose/sorbose/N-acetylgalactosamine transporter subunit IIC;	
*BFP66_RS02695*	−6.00	PTS glucose transporter subunit IIBC;	
*BFP66_RS03575*	−14.07	PTS N-acetylgalactosamine transporter subunit IIA;	
*BFP66_RS04315*	−3.47	PTS lactose/cellobiose transporter subunit IIA;	
*BFP66_RS04320*	−3.65	PTS lactose transporter subunits IICB;	
*BFP66_RS08510*	−10.66	PTS cellobiose transporter subunit IIC;	
*BFP66_RS08530*	−53.00	PTS cellbiose transporter subunit IIC;	
*BFP66_RS08570*	−5.96	PTS beta-glucoside transporter subunit EIIBCA;	
*BFP66_RS09840*	−2.24	PTS sugar transporter subunit IIC;	
*BFP66_RS09850*	−4.61	PTS lactose/cellobiose transporter subunit IIA;	
*BFP66_RS09855*	−3.09	PTS sugar transporter subunit IIB;	
*BFP66_RS05750*	−3.95	aquaporin family protein GlpF;	glycerol metabolism
*BFP66_RS05760*	−1.66	glycerol kinase GlpK;	
*BFP66_RS05755*	−3.12	type 1 glycerol-3-phosphate oxidase GlpO;	
*BFP66_RS05785*	−12.96	glycerol dehydrogenase GldA;	
*BFP66_RS05790*	−51.82	acetaldehyde/alcohol dehydrogenase;	
*BFP66_RS05800*	−2.54	Formate C-acetyltransferase/glycerol dehydratase family glycyl radical enzyme;	
*BFP66_RS09160*	−2.28	dihydroxyacetone kinase transcriptional activator DhaS;	

Note: ^a^ The threshold of *p* < 0.01 was used to identify the differentially expressed genes.

To further investigate the response of *S. sui*s to H_2_O_2_ at the transcriptional level, we selected four DEGs, namely *exo III*, *AguB*, *metQ* (encoding a methionine transporter) and *Aagp* (encoding an aquaporin), to examine their function under oxidative stress conditions. After subjecting the deletion mutations to H_2_O_2_ treatment, the survival rates of mutants Δ*Aagp*, Δ*exo III*, Δ*AguB*, and Δ*metQ* were 0.7075%, 0.0063%, 0.69%, and 9.1867%, respectively, which were significantly lower than that of wild-type (WT) strain (59.3133%) (Figure 1b). These findings suggest that these genes are involved in oxidative stress responses and support the transcriptional landscape data of *S. sui*s in response to H_2_O_2_.

### *Aagp, a novel aquaporin, is widely distributed in* S. suis

**Figure 2.**
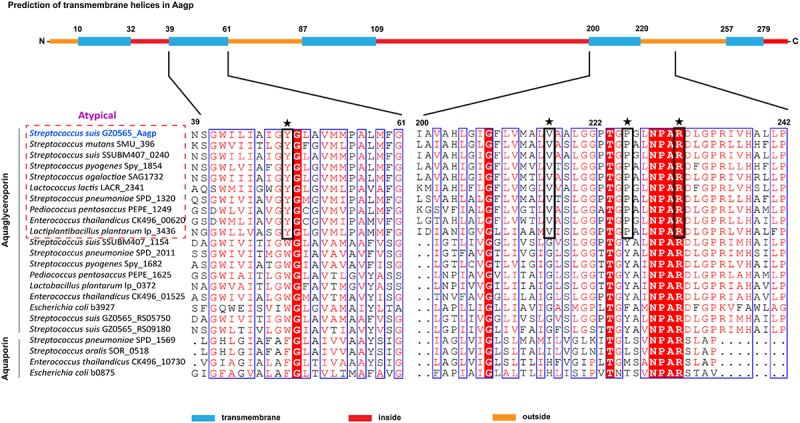
***S. suis* Aagp belongs to atypical aquaglyceroporins**. The *S. suis* Aagp transmembrane helices were predicted (top panel). Asterisks specify the ar/R region residues, and black boxes mean the atypical aquaglyceroporins’ substrate-selective residues (YVPR).

Out of the four genes associated with oxidative stress responses, *Aagp* showed the highest fold change (Table 1), making it the chosen candidate for further investigation. Aquaporins contain an aromatic/arginine (ar/R) substrate selectivity motif that comprises one arginine and three other amino acids, and these three amino acids are conserved in every aquaporin subfamily [24]. In *S. pneumoniae*, a new aquaporin subfamily called atypical aquaglyceroporin has YVPR as the substrate selectivity motif, which is different from glycerol-transporting aquaporins with WG(F/Y)R as the motif and water-transporting aquaporins with F(H/I)XR as the motif (Figure 2). *S. suis* aquaporin Aagp shares 68.17% amino acid identity with *S. pneumoniae* atypical aquaglyceroporin Pn-AqpC [25], belonging to a novel aquaporin subfamily. Aagp was predicted to be a transmembrane protein with five transmembrane loops, and it possesses substrate-selective residues YVPR as Pn-AqpC (Figure 2).

**Figure 3.**
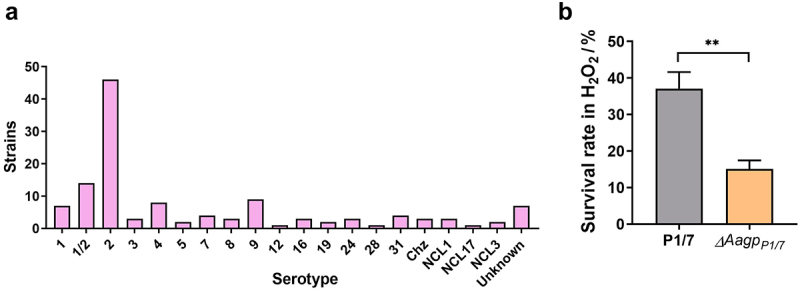
**Aagp, a novel aquaporin, is widely distributed in *S. suis***. (a) The distribution of Aagp was assessed in 126 *S. suis* strains by examining Aagp sequences obtained from the NCBI database. (b) The survival rates of P1/7 and Δ*Aagp*_*P1/7*_ were determined after 25 min treated with 25 mM H_2_O_2_. The statistical analyses were performed with a two-tailed unpaired *t* test. “**” indicate *p* < 0.01.

A search for *Aagp* in the NCBI database revealed that 126 *S. suis* strains contained *Aagp*, distributed across numerous serotypes, including serotypes 1–9, 12, 16, 19, 24, 28, and 31 (Figure 3a). To confirm the function of Aagp further, a deletion strain of *Aagp*_*P1/7*_ was constructed in *S. suis* serotype 2 strain P1/7. When challenged with H_2_O_2_, the survival rate of Δ*Aagp*_*P1/7*_ was reduced to 15.1%, compared to 37.0775% for strain P1/7 (Figure 3b). These findings provide further evidence supporting the role of *S. suis* Aagp in oxidative stress responses.

### Aagp facilitates H_2_O_2_ efflux and alleviates oxidative stress

**Figure 4.**
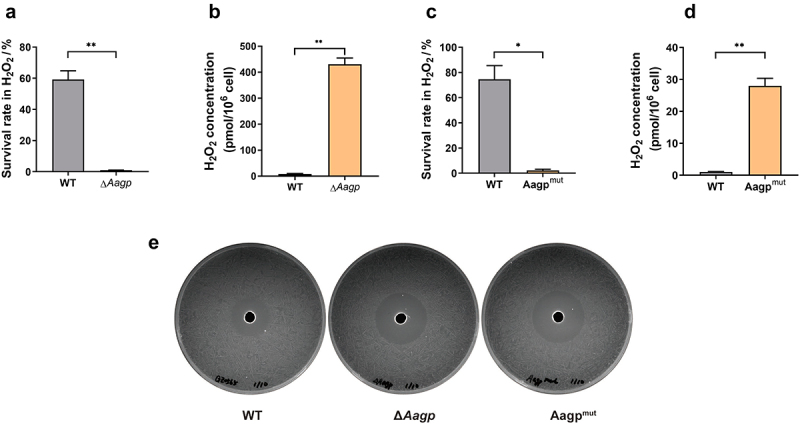
**Aagp facilitates H**_**2**_**O**_**2**_ **efflux**. (a) The survival rates of WT and Δ*Aagp* were determined after 25 min treated with 25 mM H_2_O_2_. (b) The H_2_O_2_ concentrations of WT and Δ*Aagp* were measured after 25 min treated with 25 mM H_2_O_2_. (c) The survival rates of WT and Aagp^mut^ were determined after 25 min treated with 25 mM H_2_O_2_. (d) The H_2_O_2_ concentrations of WT and Aagp^mut^ were measured after 25 min treated with 25 mM H_2_O_2_. (e) The response to H_2_O_2_ between the wild type and Aagp mutations via the Oxford cup test. The statistical analyses were performed with a two-tailed unpaired *t* test. “*” indicates *p* < 0.05 and “**” indicates *p* < 0.01.

Tong *et al*. reported that an aquaporin, So-AqpA, of *Streptococcus oligofermentans* contributes to the diffusion of H_2_O_2_ across the membrane [26]. To investigate the involvement of Aagp in H_2_O_2_ diffusion, we measured the concentration of H_2_O_2_ in bacterial cells treated with 25 mM H_2_O_2_. The concentration of H_2_O_2_ treated for 25 min in WT (59.22% survival rate) was 8.4067 pmol per 10^6^ cells, which was dramatically lower than 430.731 pmol per 10^6^ cells in Δ*Aagp* (0.96% survival rate) (Figure 4a,b). Despite several attempts, we failed to construct an *Aagp* complementary vector based on plasmids pSET1, pSET2, and pSET3. Therefore, we created a frameshift mutation strain, Aagp^mut^, by inserting one base into its CDS, as we reported in a previous study [27]. Similar to the results obtained from Δ*Aagp*, we found that the survival rate was lower in Aagp^mut^ compared with WT, while the concentration of H_2_O_2_ in Aagp^mut^ was significantly higher than in WT (Figure 4c,d). Additionally, we compared the response to H_2_O_2_ between the WT and *Aagp* mutations via the Oxford cup test. We found that both Δ*Aagp* and Aagp^mut^ strains exhibited larger inhibition zones than the WT strain (Figure 4e). These results collectively indicate that Aagp facilitates H_2_O_2_ efflux, thereby alleviating oxidative stress.

### Aagp may be involved in glycerol transport

**Figure 5.**
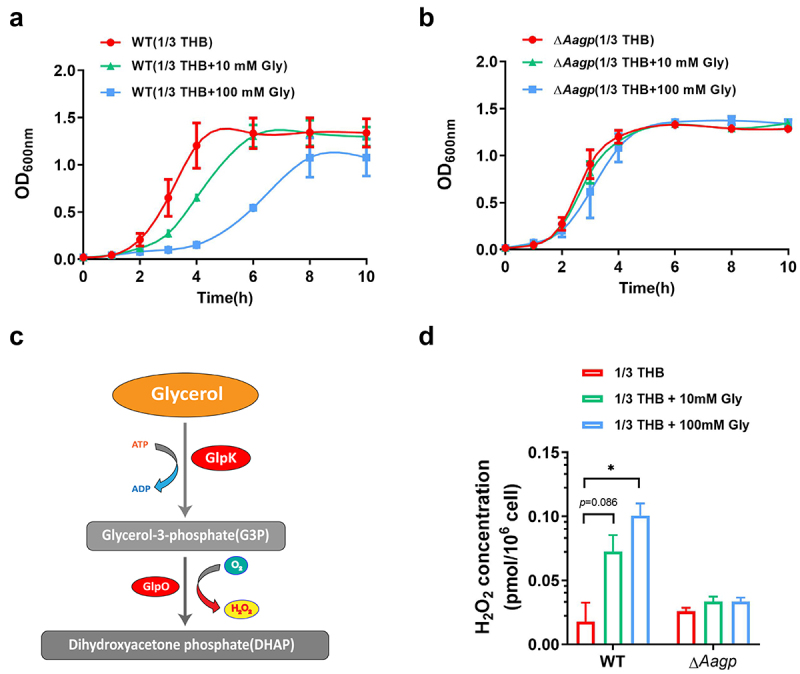
**Aagp is involved in glycerol transport**. The growth curves of WT (a) and Δ*Aagp* (b) cultured in 1/3 THB, 1/3 THB containing 10 mM glycerol or 100 mM glycerol were shown. (c) *S. suis* strain GZ0565 possesses a phosphorylation pathway for glycerol metabolism. The downregulated genes are shown in red in H_2_O_2_ treatment compared with THB condition by RNA-Seq analysis. (d) The H_2_O_2_ concentrations of WT and Δ*Aagp* cultured in 1/3 THB, 1/3 THB containing 10 mM glycerol or 100 mM glycerol were determined. The statistical analyses were performed with a two-tailed unpaired *t* test. “*” indicates *p* < 0.05.

Previous studies have shown that some aquaporins facilitate glycerol diffusion [28]. To determine the function of Aagp in relation to glycerol, the growth curves of Δ*Aagp* and WT were performed in diluted THB medium (1/3 THB as a poor medium condition) containing 10 mM glycerol or 100 mM glycerol under aerobic condition. As shown in Figure 5a, b, the growth of WT was inhibited as the concentration of glycerol increased, whereas the growth of Δ*Aagp* was not affected in the absence or presence of glycerol. As shown in Figure 5c, *S. suis* strain GZ0565 possesses genes involved in the phosphorylation pathway for glycerol metabolism, which is an energy-consuming pathway. Under the aerobic condition, glycerol may be phosphorylated by glycerol kinase (GlpK) and then oxidized by glycerol-3-phosphate oxidase (GlpO), which is accompanied by H_2_O_2_ production that can be toxic to bacteria [29]. To test this hypothesis, the concentrations of H_2_O_2_ in WT and Δ*Aagp* were measured in 1/3 THB containing 10 mM glycerol or 100 mM glycerol. The concentration of H_2_O_2_ in WT cultured in 1/3 THB was 0.0178 pmol per 10^6^ cells, but in the presence of additional glycerol, the concentration of H_2_O_2_ increased and reached to 0.1007 pmol per 10^6^ cells when cultured in 1/3 THB containing 100 mM glycerol. Conversely, in Δ*Aagp*, the concentration of H_2_O_2_ was similar in the absence or presence of glycerol (Figure 5d). These results suggest that Aagp may be involved in glycerol transport.

### *Aagp contributes to* S. suis *virulence in a mouse infection model*

**Figure 6.**
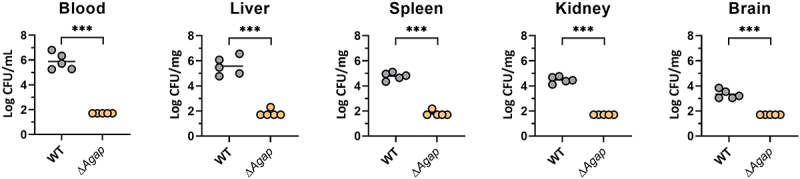
**Aagp contributes to *S. suis* virulence in a mouse infection model**. Five mice per group were injected intraperitoneally with 1.5 × 10^8^ CFU of WT and Δ*Aagp*. All mice were euthanized at 24 h post-infection. Bacteria from blood, liver, spleen, kidney and brains were plated onto THA, and colonies were expressed as Log_10_ CFU/mg or Log_10_ CFU/mL. The statistical analyses were performed with a two-tailed unpaired *t* test. “***” indicate *p* < 0.001.

Given that Aagp alleviates *S. suis* oxidative stress, the contribution of Aagp to *S. suis* virulence was assessed in mice. Compared with WT infection groups, the number of Δ*Aagp* in blood, liver, spleen, kidney, and brain tissues was significantly reduced, indicating that Aagp contributes to *S. suis* virulence (Figure 6). These results indicate that Aagp contributes to *S. suis* virulence in a mouse infection model.

## Discussion

Oxidative stress is a common challenge for *S. suis* to overcome in response to infection or environmental conditions. In this study, we employed RNA-seq to investigate the global transcriptional landscape of *S. suis* under H_2_O_2_ treatment and to reveal the adaptation mechanisms of *S. suis* response to oxidative stress. Our findings showed that multiple genes involved in oxidative stress were differentially expressed. Some of them have been identified in previous studies. We found that *BFP66_RS05715* encoding NADH oxidase, known to contribute to oxidative stress tolerance [30], was upregulated 2.30-fold by H_2_O_2_ treatment. We also observed that *BFP66_RS01615* encoding trigger factor, which contributes to stress tolerance [31], was upregulated 3.12-fold by H_2_O_2_ treatment. Additionally, we noted a downregulation of *BFP66_RS01545* encoding Fur-like protein PerR by 3.62-fold in response to H_2_O_2_ stress, consistent with previous research showing that the *PerR* deletion mutant is less sensitive to H_2_O_2_ stress [12]. PerR negatively regulates *metQ*, and its derepression increases methionine utilization, converting H_2_O_2_ to H_2_O [12]. Although RNA-Seq analysis indicated a modest 1.35-fold up-regulation of *metQ* (*BFP66_RS08225*) (*p* < 0.01), RT-qPCR analysis revealed a 10.65-fold upregulation (Supplementary Figure S2B). Moreover, compared to the WT, Δ*metQ* was more sensitive to H_2_O_2_, confirming the role of *metQ* under H_2_O_2_ conditions (Figure 1b). These findings indicate that the identified genes are crucial in *S. suis* response to oxidative stress.

Our study has uncovered novel mechanisms that may be responsible for *S. suis* resistance against oxidative stress caused by ROS. ROS can cause damage to DNA, proteins, and lipids, and repairing these molecules is crucial to combating oxidative stress. In *E. coli*, exonuclease III is a major apurinic/apyrimidinic endonuclease that recognizes base lesions and cleaves the glycosylase bond to repair DNA [32]. We found that the gene *BFP66_RS05940*, which codes for exodeoxyribonuclease III was upregulated in response to H_2_O_2_ stress, indicating that it may be involved in DNA repair through cleaving lesions. Additionally, *BFP66_RS06520* encoding an N-carbamoylputrescine amidase (AguB) was upregulated upon H_2_O_2_ stress. The AguAB pathway converts agmatine to putrescine, protecting *E. coli* from DNA damage via ROS scavenging [33]. Our deletion studies showed that exo III and AguB are crucial for H_2_O_2_ resistance in *S. suis* (Figure 1b). Furthermore, we observed upregulated genes contributing to the biosynthesis of amino acids and nucleic acid or transport, including five genes involved in producing oxidized dinucleotide cofactors (Figure 1a), *BFP66_RS01165* encoding a component of an amino acid ABC transporter, and *BFP66_RS04785* encoding a glutamate dehydrogenase. These findings indicated that *S. suis* enhances its biosynthesis and transport of amino acids and nucleic acid to respond to H_2_O_2_ stress. Interestingly, under ROS stress caused by 2,4-dichlorophenoxyacetic acid, *E. coli* reduces energy-consuming pathways to conserve energy for vital metabolism [33]. In our study, several genes related to energy-consuming pathways were downregulated under H_2_O_2_ stress (Figure 1a). In contrast, genes involved in glycolysis were upregulated, indicating that *S. suis* generates more ATP through enhanced glycolysis and reduces energy-consuming pathways for H_2_O_2_ detoxification.

The aquaporins are divided into three subfamilies: supergene channel superaquaporins, water-transporting aquaporin, and glycerol-transporting aquaglyceroporins [34–36]. Recent studies have demonstrated that aquaporins can transport H_2_O_2_ and regulate its levels in animals and plants, preventing cells from oxidative stress [37–40]. However, little is known about the function of aquaporins in H_2_O_2_ regulation in bacteria. *S. oligofermentans* aquaporin So-AqpA, sharing only 21.90% amino acid identity with *S. suis* Aagp, has been reported to facilitate the efflux of endogenous H_2_O_2_ and protect the bacterium from oxidant damage [26]. Both *S. pneumoniae* Pn-AqpC and *S. suis* Aagp are atypical aquaglyceroporins and contribute to bacterial resistance to H_2_O_2_ stress, but their mechanisms are different. Pn-AqpC can facilitate oxygen permeation into pneumococcal, which promotes the generation of endogenous H_2_O_2_ and helps *S. pneumoniae* adapt to higher exogenous H_2_O_2_ [25]. In contrast, *S. suis* Aagp contributes to resistance to oxidative stress by facilitating H_2_O_2_ efflux, similar to So-AqpA. Previous studies have shown that in *S. oligofermentans*, the expression of So-aqpA is negatively regulated by MntR and PerR [26,41]. In our RNA-seq data, we observed a significant reduction in the expressions of *MntR* (encoded by *BFP66_RS09925*) and *PerR* (encoded by *BFP66_RS01545*), accompanied by a significant upregulation of *Aagp* during H_2_O_2_ stress in *S. suis*. These findings suggest that MntR and PerR in *S. suis* may play similar roles in regulating Aagp under H_2_O_2_ stress conditions. Additionally, we propose that Aagp may possess dual functions of both H_2_O_2_ efflux and glycerol transport. In *Lactobacillus plantarum*, GlpF2, GlpF3, and GlpF4, sharing 30.37%, 36.63%, and 32.73% amino acid identity with Aagp, respectively, have been shown to be permeable to both H_2_O_2_ and glycerol [42]. In our study, we have confirmed that Aagp facilitates H_2_O_2_ efflux by oxidative stress assays and measurement of intracellular H_2_O_2_ level. However, further investigation is required to determine the glycerol transport ability of Aagp.

Through in-depth analysis, we have identified homologs of Aagp that play a role in virulence in other pathogenic bacteria. In Group B *Streptococcus*, GlpF, sharing 81% amino acid identity with Aagp, enhances the invasion capacity of Group B *Streptococcus* into eukaryotic cells [43]. In *S. pneumococci*, Pn-AqpC contributes to pneumococcal pathogenicity by modulating H_2_O_2_ production and pneumolysin release [25]. In *L. monocytogenes*, the expression of GlpF, sharing 37% amino acid identity with Aagp, is upregulated during intracellular growth [44]. When bacteria invade, host innate immune cells generate ROS such as H_2_O_2_. Neutrophils, monocytes, and macrophages, abundant in blood and tissues, play crucial roles in producing ROS to eliminate bacteria. The deletion of *Aagp* would impair the defence capability of *S. suis* survival in blood and tissues. Finally, we assume that Aagp contributes to *S. suis* oxidative stress resistance and promotes its survival in the host by facilitating H_2_O_2_ efflux.

In summary, we depicted *S. suis* defence network response to H_2_O_2_ stress and identified a novel aquaporin, Aagp, that alleviated *S. suis* oxidative stress via facilitating H_2_O_2_ efflux, which contributes to virulence. In addition, Aagp might be involved in glycerol transport.

## Supplementary Material

Supplemental Material

## Data Availability

The data supporting this study’s findings are available from the corresponding author upon reasonable request.

## References

[1] Goyette-Desjardins G, Auger JP, Xu J, et al. *Streptococcus suis*, an important pig pathogen and emerging zoonotic agent-an update on the worldwide distribution based on serotyping and sequence typing. Emerg Microbes Infect. 2014 Jun;3(6):e45. doi: 10.1038/emi.2014.45 26038745 PMC4078792

[2] Ferrando ML, de Greeff A, van Rooijen WJ, et al. Host-pathogen interaction at the intestinal mucosa correlates with zoonotic potential of *Streptococcus suis*. J Infect Dis. 2015 Jul 1;212(1):95–11. 25525050 10.1093/infdis/jiu813 PMC4462715

[3] Ferrando ML, Schultsz C. A hypothetical model of host-pathogen interaction of *Streptococcus suis* in the gastro-intestinal tract. Gut Microbes. 2016;7(2):154–162. doi: 10.1080/19490976.2016.1144008 26900998 PMC4856463

[4] Fuangthong M, Helmann JD. The OhrR repressor senses organic hydroperoxides by reversible formation of a cysteine-sulfenic acid derivative. Proc Natl Acad Sci U S A. 2002 May 14;99(10):6690–6695. 11983871 10.1073/pnas.102483199 PMC124464

[5] Imlay JA. Pathways of oxidative damage. Annu Rev Microbiol. 2003;57(1):395–418. doi: 10.1146/annurev.micro.57.030502.090938 14527285

[6] Sobota JM, Imlay JA. Iron enzyme ribulose-5-phosphate 3-epimerase in Escherichia coli is rapidly damaged by hydrogen peroxide but can be protected by manganese. Proc Natl Acad Sci U S A. 2011 Mar 29;108(13):5402–5407. 21402925 10.1073/pnas.1100410108 PMC3069151

[7] Dubbs JM, Mongkolsuk S. Peroxide-sensing transcriptional regulators in bacteria. J Bacteriol. 2012 Oct;194(20):5495–5503. doi: 10.1128/JB.00304-12 22797754 PMC3458676

[8] Imlay JA. The molecular mechanisms and physiological consequences of oxidative stress: lessons from a model bacterium. Nat Rev Microbiol. 2013 Jul;11(7):443–454. doi: 10.1038/nrmicro3032 23712352 PMC4018742

[9] Panday A, Sahoo MK, Osorio D, et al. NADPH oxidases: an overview from structure to innate immunity-associated pathologies. Cell Mol Immunol. 2015 Jan;12(1):5–23. doi: 10.1038/cmi.2014.89 25263488 PMC4654378

[10] Li H, Zhou X, Huang Y, et al. Reactive oxygen species in pathogen clearance: the killing mechanisms, the adaption response, and the side effects. Front Microbiol. 2020;11:622534. doi: 10.3389/fmicb.2020.622534 33613470 PMC7889972

[11] Willenborg J, Koczula A, Fulde M, et al. FlpS, the FNR-Like protein of *Streptococcus suis* is an essential, oxygen-sensing activator of the arginine deiminase System. Pathogens. 2016 Jul 21;5(3):51. 27455333 10.3390/pathogens5030051 PMC5039431

[12] Zhang T, Ding Y, Li T, et al. A Fur-like protein PerR regulates two oxidative stress response related operons *dpr* and *metQIN* in *Streptococcus suis*. BMC Microbiol. 2012 May 30;12(1):85. 22646062 10.1186/1471-2180-12-85 PMC3458967

[13] Hu Y, Hu Q, Wei R, et al. The XRE family transcriptional regulator SrtR in *Streptococcus suis* is involved in oxidant tolerance and virulence. Front Cell Infect Microbiol. 2018;8:452. doi: 10.3389/fcimb.2018.00452 30687648 PMC6335249

[14] Zheng C, Xu J, Li J, et al. Two spx regulators modulate stress tolerance and virulence in *Streptococcus suis* serotype 2. PLoS One. 2014;9(9):e108197. doi: 10.1371/journal.pone.0108197 25264876 PMC4180751

[15] Zhu H, Wang Y, Ni Y, et al. The redox-sensing regulator rex contributes to the virulence and oxidative stress response of *Streptococcus suis* serotype 2. Front Cell Infect Microbiol. 2018;8:317. doi: 10.3389/fcimb.2018.00317 30280091 PMC6154617

[16] Wu Z, Zhang W, Lu C. Comparative proteome analysis of secreted proteins of *Streptococcus suis* serotype 9 isolates from diseased and healthy pigs. Microb Pathog. 2008 Sep;45(3):159–166. doi: 10.1016/j.micpath.2008.04.009 18554861

[17] Wang S, Ma M, Liang Z, et al. Pathogenic investigations of *Streptococcus pasteurianus*, an underreported zoonotic pathogen, isolated from a diseased piglet with meningitis. Transbound Emerg Dis. 2022 Sep;69(5):2609–2620. doi: 10.1111/tbed.14413 34871467

[18] Wu Z, Wu C, Shao J, et al. The *Streptococcus suis* transcriptional landscape reveals adaptation mechanisms in pig blood and cerebrospinal fluid. RNA. 2014 Jun;20(6):882–898. doi: 10.1261/rna.041822.113 24759092 PMC4024642

[19] Langmead B, Salzberg SL. Fast gapped-read alignment with bowtie 2. Nat Methods. 2012 Mar 4;9(4):357–359. 22388286 10.1038/nmeth.1923 PMC3322381

[20] Robinson MD, McCarthy DJ, Smyth GK. edgeR: a bioconductor package for differential expression analysis of digital gene expression data. Bioinformatics. 2010 Jan 1;26(1):139–140. 19910308 10.1093/bioinformatics/btp616 PMC2796818

[21] Dai J, Lai L, Tang H, et al. *Streptococcus suis* synthesizes deoxyadenosine and adenosine by 5’-nucleotidase to dampen host immune responses. Virulence. 2018;9(1):1509–1520. doi: 10.1080/21505594.2018.1520544 30221577 PMC6177238

[22] Zhu Y, Dong W, Ma J, et al. Utilization of the ComRS system for the rapid markerless deletion of chromosomal genes in *Streptococcus suis*. Future Microbiol. 2019 Feb;14(3):207–222. doi: 10.2217/fmb-2018-0279 30663887

[23] Wu Z, Shao J, Ren H, et al. A *Streptococcus suis* LysM domain surface protein contributes to bacterial virulence. Vet Microbiol. 2016 May 1;187:64–69. 27066710 10.1016/j.vetmic.2016.03.017

[24] Savage DF, O’Connell JD 3rd, Miercke LJ, et al. Structural context shapes the aquaporin selectivity filter. Proc Natl Acad Sci U S A. 2010 Oct 5;107(40):17164–17169. 20855585 10.1073/pnas.1009864107 PMC2951435

[25] Hu Q, Tong H, Wang J, et al. A novel aquaporin subfamily imports oxygen and contributes to pneumococcal virulence by controlling the production and release of virulence factors. MBio. 2021 Aug 31;12(4):e0130921. 34399618 10.1128/mBio.01309-21 PMC8406300

[26] Tong H, Wang X, Dong Y, et al. A *Streptococcus* aquaporin acts as peroxiporin for efflux of cellular hydrogen peroxide and alleviation of oxidative stress. J Biol Chem. 2019 Mar 22;294(12):4583–4595. 30705089 10.1074/jbc.RA118.006877 PMC6433050

[27] Ma Z, Peng J, Yu D, et al. A streptococcal Fic domain-containing protein disrupts blood-brain barrier integrity by activating moesin in endothelial cells. PLOS Pathog. 2019 May;15(5):e1007737. doi: 10.1371/journal.ppat.1007737 31071198 PMC6529018

[28] Borgnia MJ, Agre P. Reconstitution and functional comparison of purified GlpF and AqpZ, the glycerol and water channels from *Escherichia coli*. Proc Natl Acad Sci U S A. 2001 Feb 27;98(5):2888–2893. 11226336 10.1073/pnas.051628098 PMC30235

[29] Doi Y. Glycerol metabolism and its regulation in lactic acid bacteria. Appl Microbiol Biotechnol. 2019 Jul;103(13):5079–5093. doi: 10.1007/s00253-019-09830-y 31069487

[30] Zheng C, Ren S, Xu J, et al. Contribution of NADH oxidase to oxidative stress tolerance and virulence of *Streptococcus suis* serotype 2. Virulence. 2017 Jan 2;8(1):53–65. 27315343 10.1080/21505594.2016.1201256 PMC5963199

[31] Wu T, Zhao Z, Zhang L, et al. Trigger factor of *Streptococcus suis* is involved in stress tolerance and virulence. Microb Pathog. 2011 Jul-Aug;51(1–2):69–76. doi: 10.1016/j.micpath.2010.10.001 21093574

[32] Shokolenko IN, Alexeyev MF, Robertson FM, et al. The expression of Exonuclease III from *E. coli* in mitochondria of breast cancer cells diminishes mitochondrial DNA repair capacity and cell survival after oxidative stress. DNA Repair (Amst). 2003 May 13;2(5):471–482. 12713808 10.1016/s1568-7864(03)00019-3

[33] Bhat SV, Booth SC, Vantomme EA, et al. Oxidative stress and metabolic perturbations in *Escherichia coli* exposed to sublethal levels of 2,4-dichlorophenoxyacetic acid. Chemosphere. 2015 Sep;135:453–461. 25661029 10.1016/j.chemosphere.2014.12.035

[34] Tesse A, Grossini E, Tamma G, et al. Aquaporins as targets of dietary bioactive phytocompounds. Front Mol Biosci. 2018;5:30. doi: 10.3389/fmolb.2018.00030 29721498 PMC5915544

[35] Mitchell TJ, Dalziel CE. The biology of pneumolysin. Subcell Biochem. 2014;80:145–160. 24798011 10.1007/978-94-017-8881-6_8

[36] Ishibashi K, Morishita Y, Tanaka Y. The evolutionary aspects of aquaporin family. Adv Exp Med Biol. 2017;969:35–50. 28258564 10.1007/978-94-024-1057-0_2

[37] Bienert GP, Chaumont F. Aquaporin-facilitated transmembrane diffusion of hydrogen peroxide. Biochim Biophys Acta. 2014 May;1840(5):1596–1604. doi: 10.1016/j.bbagen.2013.09.017 24060746

[38] Siefritz F, Tyree MT, Lovisolo C, et al. PIP1 plasma membrane aquaporins in tobacco: from cellular effects to function in plants. Plant Cell. 2002 Apr;14(4):869–876. doi: 10.1105/tpc.000901 11971141 PMC150688

[39] Al Ghouleh I, Frazziano G, Rodriguez AI, et al. Aquaporin 1, Nox1, and Ask1 mediate oxidant-induced smooth muscle cell hypertrophy. Cardiovasc Res. 2013 Jan 1;97(1):134–142. 22997161 10.1093/cvr/cvs295 PMC3527765

[40] Medra?O-Fernandez I, Bestetti S, Bertolotti M, et al. Stress regulates aquaporin-8 permeability to impact cell growth and survival. Antioxid Redox Signal. 2016;24(18):1031–1044. doi: 10.1089/ars.2016.6636 26972385 PMC4931348

[41] Chen Z, Wang X, Yang F, et al. Molecular insights into hydrogen peroxide-sensing mechanism of the metalloregulator mntr in controlling bacterial resistance to oxidative stresses. J Biol Chem. 2017 Mar 31;292(13):5519–5531. 28223356 10.1074/jbc.M116.764126 PMC5392694

[42] Bienert GP, Desguin B, Chaumont F, et al. Channel-mediated lactic acid transport: a novel function for aquaglyceroporins in bacteria. Biochem J. 2013 Sep 15;454(3):559–570. 23799297 10.1042/BJ20130388

[43] Johri AK, Margarit I, Broenstrup M, et al. Transcriptional and proteomic profiles of group B *Streptococcus* type V reveal potential adherence proteins associated with high-level invasion. Infect Immun. 2007 Mar;75(3):1473–1483. doi: 10.1128/IAI.00638-06 17210664 PMC1828581

[44] Monniot C, Zebre AC, Ake FM, et al. Novel listerial glycerol dehydrogenase- and phosphoenolpyruvate-dependent dihydroxyacetone kinase system connected to the pentose phosphate pathway. J Bacteriol. 2012 Sep;194(18):4972–4982. doi: 10.1128/JB.00801-12 22773791 PMC3430356

