# Supplementary material for: A novel aquaporin Aagp contributes to Streptococcus suis H2O2 efflux and virulence
Source: Virulence. 2023 Aug 24;14(1):2249789. doi: 10.1080/21505594.2023.2249789 (PMC10461500; doi:10.1080/21505594.2023.2249789)
Supplement: Supplemental Material [file KVIR_A_2249789_SM2858.zip › Supplementary Figure 1-2.docx]

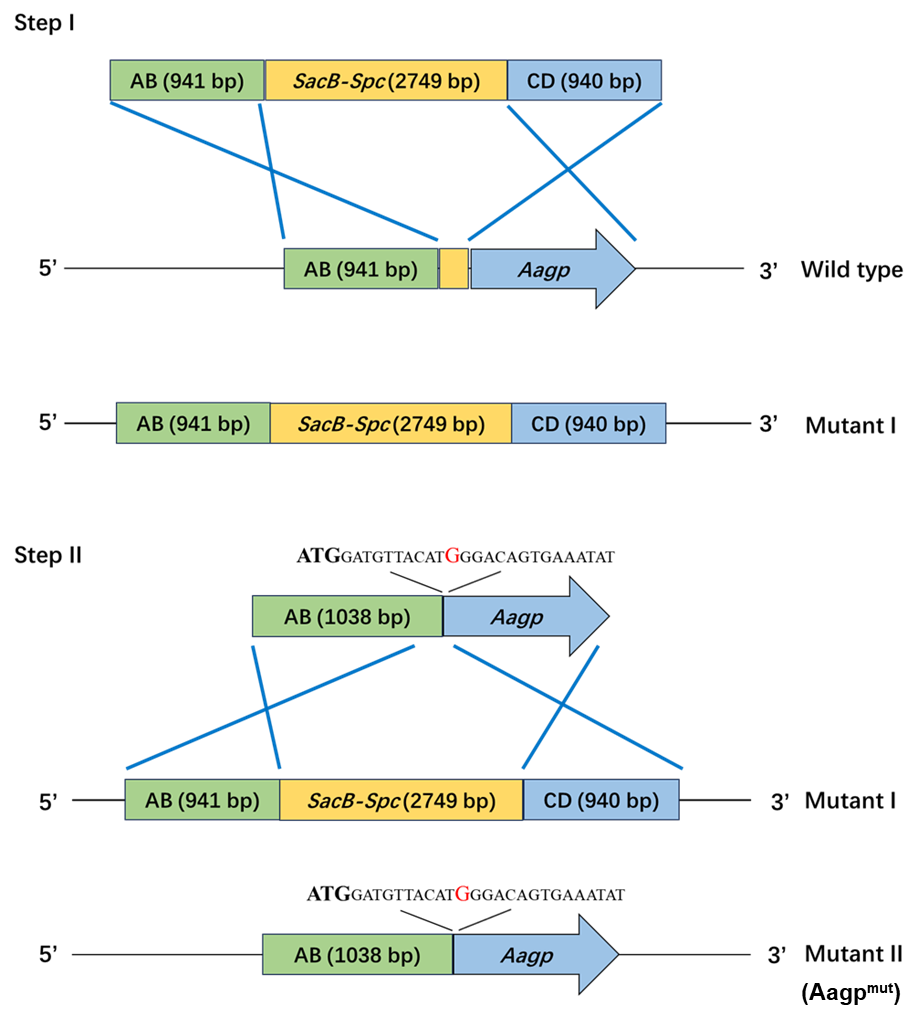


**Supplementary Figure 1.** **The construction of Aagp^mut^ through a two-step natural transformation**. Step I was performed to replace the upstream sequence of *Aagp* (marked yellow) with *SacB*-*Spc* cassette and resulted in sucrose sensitive and spectinomycin resistant. Step II was the process of cassette replacement for single base insertion via the negative selection on sucrose THA plate.


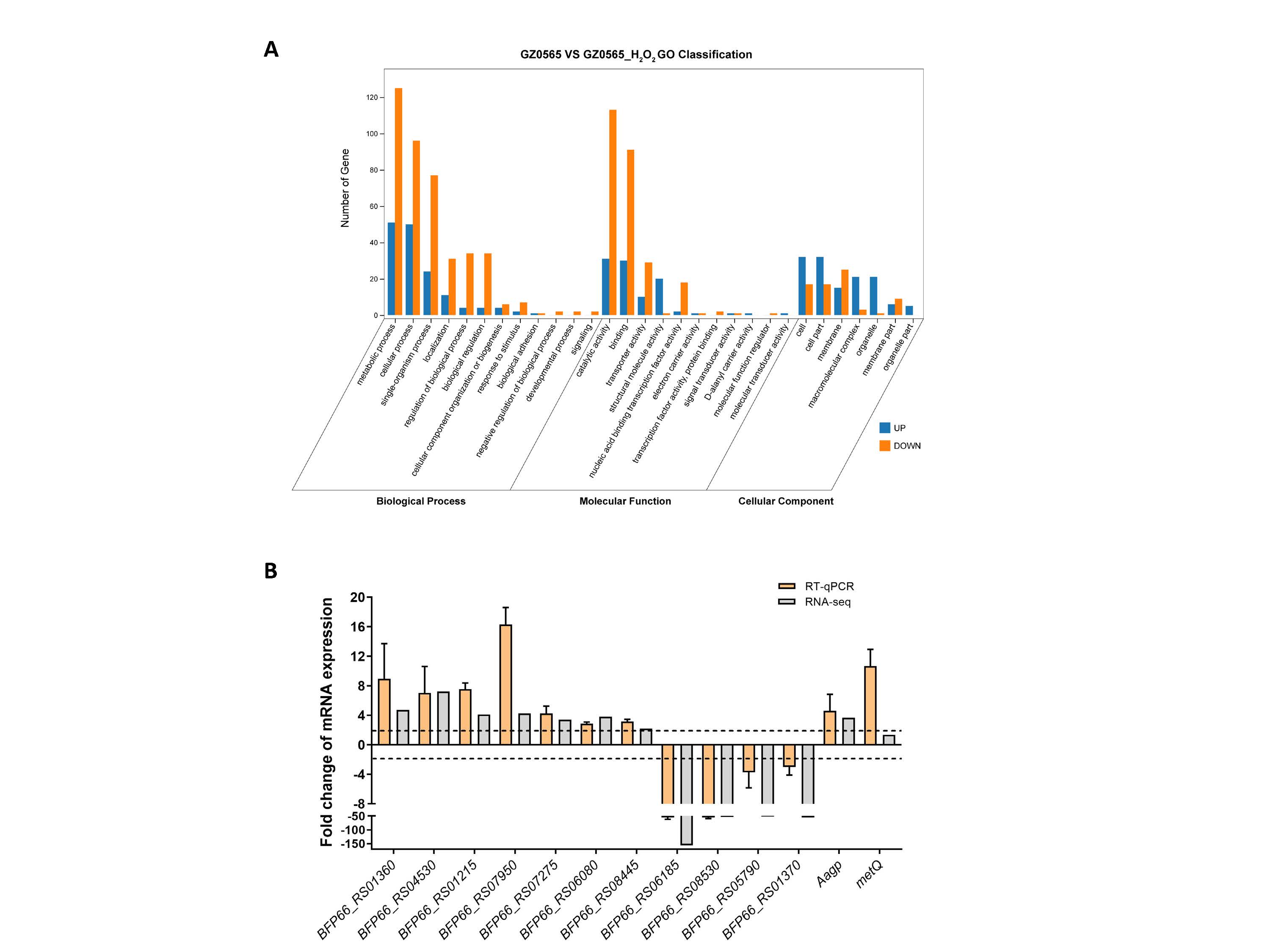


**Supplementary Figure 2** (A) The significantly enriched GO terms of the DEGs in response to 25 mM H_2_O_2_. (B) Validation of gene expression by RT-qPCR analysis.
