# Supplementary material for: A novel aquaporin Aagp contributes to Streptococcus suis H2O2 efflux and virulence
Source: Virulence. 2023 Aug 24;14(1):2249789. doi: 10.1080/21505594.2023.2249789 (PMC10461500; doi:10.1080/21505594.2023.2249789)
Supplement: Supplemental Material [file KVIR_A_2249789_SM2858.zip › Supplementary Table 1-3.docx]

**Supplementary Table 1. Bacterial strains and plasmid used in this study**

| Strains and plamids | Characteristics or sequences (5’-3') | Sources or function |
| --- | --- | --- |
| **Strains** | | |
| GZ0565 | Serotype 9 virulent strain | Serotype 9 virulent strain |
| Δ*Aagp* | The deletion mutant of *Aagp* in the background of strain GZ0565 | This study |
| Aagp^Mut^ | *Aagp* frameshift mutation strain | This study |
| Δ*exo Ⅲ* | The deletion mutant of *exo Ⅲ* in the background of strain GZ0565 | This study |
| Δ*AguB* | The deletion mutant of *AguB* in the background of strain GZ0565 | This study |
| Δ*metQ* | The deletion mutant of *metQ* in the background of strain GZ0565 | This study |
| P1/7 | Serotype 2 virulent strain | Serotype 2 virulent strain |
| Δ*Aagp_P1/7_* | The deletion mutant of *Aagp* in the background of strain P1/7 | This study |
| *E. coli* DH5α | For cloning and maintaining plasmids | TIANGEN |
| **Plasmids** | | |
| pSET4s | For constructing Δ*Aagp* and Δ*metQ* deletion mutant | This study |

**Supplementary Table 2. Primers used in this study**

| Primers | Sequences (5’-3') | Comment |
| --- | --- | --- |
| **Construction of deletion strains** | | |
| Δ*Aagp*-A | GAGTCAGAATTCGAAGACCGATTAAGAGACCAC | Upstream of fusion fragment for Δ*Aagp* |
| Δ*Aagp*-B | GAGTATTTTGTTCCTTCCA |  |
| Δ*Aagp*-C | **TGGAAGGAACAAAATACTC**TATAGAGAACCCGTTTCGTCG | Downstream of fusion fragment for Δ*Aagp* |
| Δ*Aagp*-D | CGCGGATCCACAACTGAGTAGCTTGAAGGACGTT |  |
| pSET4s-F | GCACAGATGCGTAAGGAGAA | Detection of inserted fragments of pSET4s |
| pSET4s-R | TTCCGGCTCGTATGTTGTGT |  |
| Δ*Aagp*-F | TACATGGACAGTGAAATA | Detection of deletion of *Aagp* gene |
| Δ*Aagp*-R | AATTGATGCGATAATTGG |  |
| *SacB-Spc*-F | GGATAATGCTGAAAACTCCTT | *SacB*-*Spc* gene cassette |
| *SacB-Spc*-R | AATCTGATTACCAATTAGAATGAATAT |  |
| Aagp^Mut^-A | TGTAAACTGGTATATAGTTCCCTA | Upstream of fusion fragment for Aagp^Mut^ mutant Ⅰ |
| Aagp^Mut^-B | **AAGGAGTTTTCAGCATTATCC** AGTAAACCATTTTTAGAAAGTAAACG |  |
| Aagp^Mut^-C | **ATATTCATTCTAATTGGTAATCAGATT** ATGGATGTTACATGGACAGTGA | Downstream of fusion fragment for Aagp^Mut^ mutant Ⅰ |
| Aagp^Mut^-D | GTAGCATAACTTTTAGGAAAATAG |  |
| Aagp^Mut^-A | TGTAAACTGGTATATAGTTCCCTA | Upstream of fusion fragment for Aagp^Mut^ mutant Ⅱ |
| Aagp^Mut^-R | ATATTTCACTGTCCCATGTAACATCCAT |  |
| Aagp^Mut^-C1 | ATGGATGTTACATGGGACAGTGAAATAT | Downstream of fusion fragment for Aagp^Mut^ mutant Ⅱ |
| Aagp^Mut^-D | GTAGCATAACTTTTAGGAAAATAG |  |
| Aagp^Mut^-E | GATAATCCCCTTTTTCATATTT | Detection of Aagp^Mut^ |
| Aagp^Mut^-F | CTATTTTCCTAAAAGTTATGCTAC |  |
| *Spc*-F | ACTAGTGTTCGTGAATACATGTT | Spectinomycin resistance gene |
| *Spc*-R | AATCTGATTACCAATTAGAATGAATAT |  |
| Δ*exo Ⅲ*-A | TTCTGCCTTTCCTGTTGT | Upstream of fusion fragment for Δ*exo Ⅲ* |
| Δ*exo Ⅲ*-B | TTCACGAACACTAGTAATTTTCCTTTTCTAGTC |  |
| Δ*exo Ⅲ*-C | ATTGGTAATCAGATTCAGTGGATATTAATACTC | Downstream of fusion fragment for Δ*exo Ⅲ* |
| Δ*exo Ⅲ*-D | TGCACCTGAAATGAGTCC |  |
| Δ*exo Ⅲ*-A1 | GTAGTAACCAATCCCATCTTGA | Fusion fragment for Δ*exo Ⅲ* |
| Δ*exo Ⅲ*-D1 | ACCTTTTGCTTCTGCTAATATG |  |
| Δ*exo Ⅲ*-F | TGTTACTGCATCCAATACAATAGCAC | Detection of deletion of *exo Ⅲ* gene |
| Δ*exo Ⅲ*-R | ATTGCTATCTTCCTTTAAGTAAGGCT |  |
| Δ*AguB*-A | CCGGAATTCTTTACATCTGCCGATGAGTTGC | Upstream of fusion fragment for Δ*AguB* |
| Δ*AguB*-B | TATCAGCAGATTGTAGAATAAACA |  |
| Δ*AguB*-C | TACAATCTGCTGATATGCTACAGTAACATTTCTCATA | Downstream of fusion fragment for Δ*AguB* |
| Δ*AguB*-D | TGCACTGCAGGTGGCTAGTCGATTTAGTCAGG |  |
| Δ*AguB*-X | CAGTTTCCTCCTCACGTCCA | Detection of deletion of *AguB* gene |
| Δ*AguB*-Y | GAGTTTACCGCAAGACCCAT |  |
| Δ*AguB*-F | ATTTCAATCGGTAGTATCATCC | Detection of deletion of *AguB* gene |
| Δ*AguB*-R | TCTTTATGGGGCTAAATGTCC |  |
| Δ*metQ*-A | CCGGAATTCCCATAGGAAGAATGAATATCCA | Upstream of fusion fragment for Δ*metQ* |
| Δ*metQ*-B | TAAAATGTAAATAGGTTGAGGACT |  |
| Δ*metQ*-C | **AGTCCTCAACCTATTTACATTTTA**CATAAAATGATCTCCTTAAATTAG | Downstream of fusion fragment for Δ*metQ* |
| Δ*metQ*-D | TCCCCCGGGATGGGAAAAGTTATCATTGGAATT |  |
| Δ*metQ*-X | TCCATACCGTCTGAAGTCTC | Detection of deletion of *metQ* gene |
| Δ*metQ*-Y | TACTTATATCGCACCAATCCG |  |
| Δ*metQ*-M | AATTGAACTTTACTAGCTGCC | Detection of deletion of *metQ* gene |
| Δ*metQ*-N | GTGAAACCTAAAAGCCGAA |  |
| Δ*metQ*-F | GCCTTCAATCCTAATCGATGGCTCAA | Detection of deletion of *metQ* gene |
| Δ*metQ*-R | TTTTTATGCTGGCTCCAGTATAGTTT |  |
| Δ*Aagp_P1/7_*-A | AAAAGAGGAAATTGCTTAGTAC | Upstream of fusion fragment for Δ*Aagp_P1/7_*- |
| Δ*Aagp_P1/7_*-B | TTCACGAACACTAGTATTTTTCTTATATTTCCTTTCA |  |
| Δ*Aagp_P1/7_*-C | ATTGGTAATCAGATTTATAGAGAACCCGTTTCGTCGG | Downstream of fusion fragment for Δ*Aagp_P1/7_*- |
| Δ*Aagp_P1/7_*-D | CAAGACTTCATAGAGTTCTGAT |  |
| Δ*Aagp_P1/7_*-A1 | GAGAAGAGTGAAAAGTTCGCAA | Upstream of fusion fragment for Δ*Aagp_P1/7_*- |
| Δ*Aagp_P1/7_*-D1 | AATAGCTGAGTAGCTTGAAGGA | Downstream of fusion fragment for Δ*Aagp_P1/7_*- |
| Δ*Aagp_P1/7_*-F | CTGTTGGTCTTGCTTTGGGAGGAA | Detection of deletion of *Aagp_P1/7_*- gene |
| Δ*Aagp_P1/7_*-R | GTGTAAAGGCGCGAAGACCATGTT |  |
|  |  |  |
| **RT-qPCR** | | |
| *qRS01215*-F | GGCCAACCTTTCTCCCAGAT | The transcriptional level of *RS01215* mRNA |
| *qRS01215*-R | TATCCCCCACAGCGTCTACA |  |
| *qRS05620*-F | TGACACGGATCAAACCTTCTAC | The transcriptional level of *RS05620* (*ParC*) mRNA |
| *qRS05620*-R | TCCCAACAGGTGCCATTATC |  |
| *qAagp*-F | CGTAAAGCTGCGACAACTAATG | The transcriptional level of *Aagp* mRNA |
| *qAagp*-R | TACACCAACCCAAGCATACC |  |
| *qRS01360*-F | CAGCCTTAGCGATTTTACGT | The transcriptional level of *RS01360* mRNA |
| *qRS01360*-R | AAGTAGCAGCTCCAACTGTG |  |
| *qRS01370*-F | CTAGTCCCATAGCACCGAAG | The transcriptional level of *RS01370* mRNA |
| *qRS01370*-R | TTCCAAAGTAGCCTTCAACC |  |
| *qRS04530*-F | CATGGTAGACGATTACCCAGAAG | The transcriptional level of *RS04530* mRNA |
| *qRS04530*-R | CATCGGAAGCGTGACAACTA |  |
| *qRS05790*-F | GCTCATCCAACAAATCCTCTCT | The transcriptional level of *RS05790* mRNA |
| *qRS05790*-R | GACTTCACCGTTTACCACTCTAC |  |
| *qRS06080*-F | CATCAACTCCACAACACCAG | The transcriptional level of *RS06080* mRNA |
| *qRS06080*-R | GATGAAGACCTATTTGATGC |  |
| *qRS06185*-F | AATAAATTTTGTCAATGTCT | The transcriptional level of *RS06185* mRNA |
| *qRS06185*-R | TGGTCAAGCTCGTCTAACCC |  |
| *qRS07275*-F | AAAATAGCCATGAGACTCTG | The transcriptional level of *RS07275* mRNA |
| *qRS07275*-R | AATCCAGCAGATAAGCGACA |  |
| *qRS07950*-F | TTGACAGGCGTTGCTGGTAT | The transcriptional level of *RS07950* mRNA |
| *qRS07950*-R | GACCACCGACAACCCAGTAG |  |
| *qRS08445*-F | TGAGCTTGTCGAGGTTGATG | The transcriptional level of *RS08445* mRNA |
| *qRS08445*-R | AATAACCTCTGGTTGACCCAC |  |
| *qRS08530*-F | TTATCCAAGACCAATTTAGC | The transcriptional level of *RS08530* mRNA |
| *qRS08530*-R | GTACTTAATTAGCCCGCAGA |  |
| *qmetQ-F* | CTTGGCATTGGATGTTTGG | The transcriptional level of *metQ* mRNA |
| *qmetQ-R* | ACCTTGTCCCAGTTGATTACTT |  |

^a^ Bolded nucleotides denote reverse complement; underlined nucleotides denote restriction enzyme sites.

**Supplementary Table 3. Genes differentially expressed at the transcriptional level in GZ0565 exposed to 25 mM H_2_O_2_**

| ID | FC (H_2_O_2_/THB) | P Value | Product |
| --- | --- | --- | --- |
| *BFP66_RS04530* | 7.22 | 0.000195594 | glucan-binding protein; |
| *BFP66_RS01360* | 4.73 | 0.001396375 | 50S ribosomal protein L32; |
| *BFP66_RS07950* | 4.27 | 0 | membrane protein insertase YidC; |
| *BFP66_RS01215* | 4.10 | 0 | hypothetical protein; |
| *BFP66_RS06080* | 3.82 | 0.000411758 | D-alanine--poly(phosphoribitol) ligase subunit 2; |
| *BFP66_RS08925* | 3.80 | 2.02E-49 | DUF1761 domain-containing protein; |
| *BFP66_RS03035* | 3.68 | 0 | 50S ribosomal protein L1; |
| *BFP66_RS01250* | 3.66 | 0 | aquaporin family protein Aagp; |
| *BFP66_RS02310* | 3.62 | 2.35E-28 | DUF3165 domain-containing protein; |
| *BFP66_RS09695* | 3.57 | 0.000632488 | hypothetical protein; |
| *BFP66_RS07275* | 3.41 | 0.000025 | MarR family transcriptional regulator; |
| *BFP66_RS05460* | 3.37 | 0.00092488 | hypothetical protein; |
| *BFP66_RS03030* | 3.26 | 0 | 50S ribosomal protein L11; |
| *BFP66_RS01345* | 3.25 | 0.000000107 | transcriptional regulator; |
| *BFP66_RS01220* | 3.22 | 3.87E-264 | DUF1700 domain-containing protein; |
| *BFP66_RS01245* | 3.16 | 5.03E-291 | formate/nitrite transporter; |
| *BFP66_RS08470* | 3.15 | 4.45E-89 | 30S ribosomal protein S18; |
| *BFP66_RS01615* | 3.12 | 0 | trigger factor; |
| *BFP66_RS04920* | 3.11 | 0 | 50S ribosomal protein L7/L12; |
| *BFP66_RS06425* | 3.10 | 0.001893535 | IS110 family transposase; |
| *BFP66_RS01665* | 3.07 | 0 | fructose-bisphosphate aldolase; |
| *BFP66_RS05940* | 2.89 | 4.04E-229 | exodeoxyribonuclease III; |
| *BFP66_RS06400* | 2.86 | 1.07E-208 | MBL fold metallo-hydrolase; |
| *BFP66_RS02590* | 2.86 | 0 | triose-phosphate isomerase; |
| *BFP66_RS02970* | 2.80 | 5.94E-83 | peptidylprolyl isomerase; |
| *BFP66_RS08875* | 2.77 | 0.000252965 | hypothetical protein; |
| *BFP66_RS09135* | 2.77 | 0.005164626 | GlsB/YeaQ/YmgE family stress response membrane protein; |
| *BFP66_RS01900* | 2.76 | 4.93E-124 | glycogen/starch/alpha-glucan family phosphorylase; |
| *BFP66_RS01225* | 2.75 | 8.25E-17 | PadR family transcriptional regulator; |
| *BFP66_RS08180* | 2.71 | 2.89E-31 | hypothetical protein; |
| *BFP66_RS07955* | 2.69 | 3.38E-149 | transcription elongation factor GreA; |
| *BFP66_RS01475* | 2.68 | 0 | molecular chaperone DnaJ; |
| *BFP66_RS07270* | 2.67 | 0.000000132 | ABC transporter ATP-binding protein; |
| *BFP66_RS05680* | 2.67 | 9.1E-129 | VOC family protein; |
| *BFP66_RS09265* | 2.63 | 0 | elongation factor Ts; |
| *BFP66_RS07495* | 2.61 | 0 | 2,3-bisphosphoglycerate-dependent phosphoglycerate mutase; |
| *BFP66_RS04160* | 2.61 | 0 | L-lactate dehydrogenase LDH; |
| *BFP66_RS08475* | 2.59 | 0 | single-stranded DNA-binding protein; |
| *BFP66_RS01670* | 2.59 | 2.42E-27 | 50S ribosomal protein L28; |
| *BFP66_RS10180* | 2.56 | 0 | 30S ribosomal protein S4; |
| *BFP66_RS07005* | 2.56 | 1.06E-251 | branched-chain amino acid ABC transporter substrate-binding protein; |
| *BFP66_RS10225* | 2.55 | 1.54E-67 | nuclear transport factor 2 family protein; |
| *BFP66_RS03415* | 2.55 | 1.03E-96 | ABC transporter ATP-binding protein; |
| *BFP66_RS04785* | 2.53 | 1.02E-98 | amino acid ABC transporter substrate-binding protein; |
| *BFP66_RS07375* | 2.52 | 3.19E-39 | queuosine transporter QueT; |
| *BFP66_RS08900* | 2.50 | 0 | 30S ribosomal protein S9; |
| *BFP66_RS03340* | 2.48 | 0.0000621 | 50S ribosomal protein L35; |
| *BFP66_RS08125* | 2.46 | 1.53E-66 | 1,4-dihydroxy-2-naphthoate polyprenyltransferase; |
| *BFP66_RS03345* | 2.46 | 0 | 50S ribosomal protein L20; |
| *BFP66_RS07265* | 2.45 | 2.92E-31 | glycine/betaine ABC transporter permease; |
| *BFP66_RS04205* | 2.44 | 4.47E-286 | ribose-phosphate diphosphokinase; |
| *BFP66_RS10620* | 2.44 | 4.9E-76 | topoisomerase; |
| *BFP66_RS05330* | 2.43 | 2E-79 | KH domain-containing protein; |
| *BFP66_RS01350* | 2.43 | 4.4E-15 | cadmium transporter; |
| *BFP66_RS02510* | 2.43 | 1.11E-14 | hypothetical protein; |
| *BFP66_RS00350* | 2.40 | 1.73E-55 | 30S ribosomal protein S17; |
| *BFP66_RS07260* | 2.39 | 2.22E-17 | polyketide cyclase; |
| *BFP66_RS06465* | 2.38 | 0 | Mac family protein; |
| *BFP66_RS05530* | 2.37 | 0.0000605 | hypothetical protein; |
| *BFP66_RS06865* | 2.36 | 0 | enolase; |
| *BFP66_RS02305* | 2.35 | 3.57E-286 | translational GTPase TypA; |
| *BFP66_RS07855* | 2.34 | 2.1E-297 | ECF transporter S component; |
| *BFP66_RS00400* | 2.33 | 0.00035693 | 50S ribosomal protein L30; |
| *BFP66_RS09795* | 2.33 | 0 | Gamma-glutamyltranspeptidase; |
| *BFP66_RS09570* | 2.31 | 3.94E-206 | glutamate--tRNA ligase; |
| *BFP66_RS05715* | 2.30 | 0 | NADH oxidase; |
| *BFP66_RS08870* | 2.29 | 6.76E-15 | otitis media-associated H10; |
| *BFP66_RS03410* | 2.28 | 2.5E-61 | ABC transporter permease; |
| *BFP66_RS08130* | 2.25 | 1E-55 | FAD:protein FMN transferase; |
| *BFP66_RS05195* | 2.25 | 0.000000219 | hypothetical protein; |
| *BFP66_RS01165* | 2.22 | 1.6E-83 | NADP-specific glutamate dehydrogenase; |
| *BFP66_RS03605* | 2.22 | 6.75E-96 | hyaluronidase; |
| *BFP66_RS08445* | 2.21 | 0 | cell surface protein; |
| *BFP66_RS06935* | 2.21 | 2.57E-54 | flavodoxin; |
| *BFP66_RS08480* | 2.20 | 1.04E-178 | 30S ribosomal protein S6; |
| *BFP66_RS00310* | 2.19 | 0 | 50S ribosomal protein L4; |
| *BFP66_RS09820* | 2.19 | 0 | amino acid ABC transporter substrate-binding protein; |
| *BFP66_RS06470* | 2.19 | 0 | pyruvate kinase; |
| *BFP66_RS06160* | 2.19 | 5.46E-09 | N-acetyl-beta-hexosaminidase; |
| *BFP66_RS08440* | 2.18 | 3.04E-214 | hypothetical protein; |
| *BFP66_RS05335* | 2.18 | 2.71E-21 | 30S ribosomal protein S16; |
| *BFP66_RS05930* | 2.17 | 2.44E-116 | amino acid permease; |
| *BFP66_RS05560* | 2.17 | 1.28E-159 | tRNA 4-thiouridine(8) synthase ThiI; |
| *BFP66_RS08435* | 2.17 | 9.84E-53 | hypothetical protein; |
| *BFP66_RS08135* | 2.16 | 2.65E-98 | FMN-binding protein; |
| *BFP66_RS09075* | 2.16 | 1.1E-225 | purine permease; |
| *BFP66_RS00790* | 2.16 | 0 | type I glyceraldehyde-3-phosphate dehydrogenase; |
| *BFP66_RS03405* | 2.14 | 1.12E-63 | ABC transporter substrate-binding protein; |
| *BFP66_RS02585* | 2.14 | 0 | elongation factor Tu; |
| *BFP66_RS01335* | 2.14 | 0.003405379 | arsenate reductase (glutaredoxin); |
| *BFP66_RS00115* | 2.14 | 0.000303366 | acyl carrier protein; |
| *BFP66_RS00355* | 2.13 | 1.47E-205 | 50S ribosomal protein L14; |
| *BFP66_RS08275* | 2.11 | 5.07E-160 | serine--tRNA ligase; |
| *BFP66_RS00585* | 2.10 | 0.0000104 | hypothetical protein; |
| *BFP66_RS05510* | 2.10 | 1.52E-25 | hypothetical protein; |
| *BFP66_RS07745* | 2.09 | 8.61E-81 | nicotinate phosphoribosyltransferase; |
| *BFP66_RS06050* | 2.08 | 4.6E-61 | iron ABC transporter permease; |
| *BFP66_RS04210* | 2.07 | 7.82E-18 | CYTH domain-containing protein; |
| *BFP66_RS05590* | 2.07 | 0 | 30S ribosomal protein S1; |
| *BFP66_RS05240* | 2.06 | 1.84E-28 | aspartate aminotransferase; |
| *BFP66_RS06535* | 2.05 | 1.06E-200 | saccharopine dehydrogenase; |
| *BFP66_RS00375* | 2.05 | 1.91E-177 | 30S ribosomal protein S8; |
| *BFP66_RS04915* | 2.05 | 3.98E-277 | 50S ribosomal protein L10; |
| *BFP66_RS00340* | 2.04 | 1.39E-281 | 50S ribosomal protein L16; |
| *BFP66_RS08780* | 2.03 | 1.33E-215 | glutamine ABC transporter substrate-binding protein; |
| *BFP66_RS09270* | 2.03 | 0 | 30S ribosomal protein S2; |
| *BFP66_RS02985* | 2.02 | 7.98E-36 | hypothetical protein; |
| *BFP66_RS01580* | 2.02 | 0.000000115 | NADP-dependent oxidoreductase; |
| *BFP66_RS00780* | 2.02 | 0 | elongation factor G; |
| *BFP66_RS08280* | 2.01 | 0.0011878 | PspC domain-containing protein; |
| *BFP66_RS06520* | 2.01 | 6.02E-123 | N-carbamoylputrescine amidase; |
| *BFP66_RS03400* | 2.00 | 1.56E-36 | ABC transporter substrate-binding protein; |
| *BFP66_RS02700* | -2.01 | 0.0000883 | MurR/RpiR family transcriptional regulator; |
| *BFP66_RS05025* | -2.01 | 0.000000854 | putative DNA-binding protein; |
| *BFP66_RS00180* | -2.02 | 4.81E-92 | hypothetical protein; |
| *BFP66_RS10695* | -2.02 | 3.62E-11 | transposase; |
| *BFP66_RS10680* | -2.02 | 3.7E-11 | transposase; |
| *BFP66_RS10515* | -2.02 | 3.72E-11 | transposase; |
| *BFP66_RS10475* | -2.02 | 3.75E-11 | transposase; |
| *BFP66_RS10800* | -2.02 | 3.82E-11 | transposase; |
| *BFP66_RS10900* | -2.02 | 3.9E-11 | transposase; |
| *BFP66_RS10850* | -2.02 | 3.92E-11 | transposase; |
| *BFP66_RS10790* | -2.02 | 3.93E-11 | transposase; |
| *BFP66_RS04510* | -2.02 | 0.000574756 | radical SAM protein; |
| *BFP66_RS05265* | -2.04 | 1.66E-57 | endonuclease III; |
| *BFP66_RS04515* | -2.04 | 0.00000321 | ABC transporter ATP-binding protein; |
| *BFP66_RS08810* | -2.04 | 4.74E-37 | acetolactate synthase small subunit; |
| *BFP66_RS03580* | -2.04 | 0.0000146 | glycosyl hydrolase family 88; |
| *BFP66_RS00860* | -2.05 | 1.26E-27 | alpha-galactosidase; |
| *BFP66_RS00930* | -2.05 | 0.006321301 | transketolase; |
| *BFP66_RS05670* | -2.05 | 2.59E-11 | ABC transporter ATP-binding protein; |
| *BFP66_RS07985* | -2.06 | 1.36E-169 | hypothetical protein; |
| *BFP66_RS10875* | -2.06 | 4.16E-15 | DUF454 domain-containing protein; |
| *BFP66_RS00995* | -2.06 | 4.89E-38 | DNA polymerase IV; |
| *BFP66_RS00440* | -2.06 | 0 | DNA-directed RNA polymerase subunit alpha; |
| *BFP66_RS04795* | -2.07 | 6.18E-125 | glycogen synthase GlgA; |
| *BFP66_RS06615* | -2.07 | 0 | glycogen/starch/alpha-glucan family phosphorylase; |
| *BFP66_RS09905* | -2.07 | 1.25E-218 | metal ABC transporter ATP-binding protein; |
| *BFP66_RS10290* | -2.07 | 1.05E-14 | DNA replication and repair protein RecF; |
| *BFP66_RS02680* | -2.07 | 3.66E-15 | haloacid dehalogenase; |
| *BFP66_RS02950* | -2.09 | 7.85E-216 | UDP-N-acetylmuramoyl-tripeptide--D-alanyl-D- alanine ligase; |
| *BFP66_RS01200* | -2.10 | 8.52E-49 | ABC transporter ATP-binding protein; |
| *BFP66_RS01260* | -2.10 | 0.003404078 | CoA pyrophosphatase; |
| *BFP66_RS04900* | -2.10 | 0.0000912 | sugar O-acetyltransferase; |
| *BFP66_RS10365* | -2.10 | 4.77E-44 | hypothetical protein; |
| *BFP66_RS10410* | -2.11 | 1.76E-127 | serine hydrolase; |
| *BFP66_RS10770* | -2.13 | 2.28E-23 | restriction endonuclease subunit M; |
| *BFP66_RS08495* | -2.13 | 1.57E-24 | histidine phosphatase family protein; |
| *BFP66_RS01235* | -2.14 | 0.0000527 | IS1595 family transposase; |
| *BFP66_RS08560* | -2.14 | 6.24E-53 | AraC family transcriptional regulator; |
| *BFP66_RS02245* | -2.15 | 9.83E-11 | glyoxalase/bleomycin resistance protein/dioxygenase; |
| *BFP66_RS01760* | -2.15 | 3.29E-84 | cysteine hydrolase; |
| *BFP66_RS02960* | -2.17 | 0 | ATPase; |
| *BFP66_RS06460* | -2.17 | 0 | FAD-binding oxidoreductase; |
| *BFP66_RS00945* | -2.17 | 8.59E-16 | SIS domain-containing protein; |
| *BFP66_RS09920* | -2.18 | 0 | metal transporter; |
| *BFP66_RS09485* | -2.19 | 0.0000278 | type II toxin-antitoxin system Phd/YefM family antitoxin; |
| *BFP66_RS08945* | -2.20 | 2.53E-37 | DNA-binding response regulator; |
| *BFP66_RS08960* | -2.20 | 0.005024676 | sugar ABC transporter substrate-binding protein; |
| *BFP66_RS03180* | -2.21 | 4.58E-11 | ArsR family transcriptional regulator; |
| *BFP66_RS00850* | -2.22 | 1.92E-15 | sugar ABC transporter permease; |
| *BFP66_RS10340* | -2.23 | 8.17E-09 | 23S rRNA (pseudouridine(1915)-N(3))-methyltransferase RlmH; |
| *BFP66_RS09840* | -2.24 | 2.36E-28 | PTS sugar transporter subunit IIC; |
| *BFP66_RS02655* | -2.25 | 3.16E-119 | ferrous iron transport protein B; |
| *BFP66_RS06235* | -2.27 | 1.1E-75 | peptidase; |
| *BFP66_RS09160* | -2.28 | 1.86E-22 | dihydroxyacetone kinase transcriptional activator DhaS; |
| *BFP66_RS01610* | -2.30 | 7.46E-55 | alpha/beta hydrolase; |
| *BFP66_RS02165* | -2.33 | 0.003195189 | ISL3 family transposase; |
| *BFP66_RS08995* | -2.34 | 1.14E-17 | alpha-mannosidase; |
| *BFP66_RS04400* | -2.34 | 0.005354759 | N-acetyltransferase; |
| *BFP66_RS02465* | -2.34 | 5.61E-72 | hypothetical protein; |
| *BFP66_RS05070* | -2.34 | 9.05E-46 | L-allo-threonine dehydrogenase; |
| *BFP66_RS09530* | -2.35 | 2.2E-91 | argininosuccinate lyase; |
| *BFP66_RS00845* | -2.35 | 4.33E-54 | sugar ABC transporter substrate-binding protein; |
| *BFP66_RS05050* | -2.35 | 0.000171092 | hypothetical protein; |
| *BFP66_RS08895* | -2.37 | 3.68E-13 | integrase; |
| *BFP66_RS01740* | -2.38 | 0.00000251 | IS200/IS605 family transposase; |
| *BFP66_RS06095* | -2.38 | 0.00000256 | IS200/IS605 family transposase; |
| *BFP66_RS06250* | -2.38 | 0.00000258 | IS200/IS605 family transposase; |
| *BFP66_RS04420* | -2.38 | 0.00000261 | IS200/IS605 family transposase; |
| *BFP66_RS01770* | -2.38 | 0.00000263 | IS200/IS605 family transposase; |
| *BFP66_RS02170* | -2.38 | 0.00000264 | IS200/IS605 family transposase; |
| *BFP66_RS04100* | -2.38 | 0.00000267 | IS200/IS605 family transposase; |
| *BFP66_RS01660* | -2.38 | 0.0000027 | IS200/IS605 family transposase; |
| *BFP66_RS04290* | -2.38 | 0.003569964 | galactose-6-phosphate isomerase subunit LacA; |
| *BFP66_RS02375* | -2.38 | 1.08E-96 | DUF1827 domain-containing protein; |
| *BFP66_RS05575* | -2.38 | 0.0000154 | hypothetical protein; |
| *BFP66_RS01110* | -2.39 | 9.62E-192 | thioredoxin; |
| *BFP66_RS00725* | -2.39 | 6.43E-29 | DUF4651 domain-containing protein; |
| *BFP66_RS09280* | -2.40 | 0 | serine protease; |
| *BFP66_RS06485* | -2.41 | 5.11E-34 | GntR family transcriptional regulator; |
| *BFP66_RS00885* | -2.42 | 2.89E-18 | ABC transporter ATP-binding protein; |
| *BFP66_RS04990* | -2.42 | 8.15E-31 | histidine kinase; |
| *BFP66_RS00855* | -2.45 | 1.48E-12 | carbohydrate ABC transporter permease; |
| *BFP66_RS08935* | -2.46 | 0 | ABC transporter ATP-binding protein; |
| *BFP66_RS06005* | -2.46 | 3.54E-49 | hypothetical protein; |
| *BFP66_RS07830* | -2.47 | 0.0000643 | CsbD family protein; |
| *BFP66_RS09615* | -2.48 | 1.07E-70 | hypothetical protein; |
| *BFP66_RS08500* | -2.49 | 8.09E-31 | histidine phosphatase family protein; |
| *BFP66_RS09960* | -2.49 | 0.000000632 | IS110 family transposase; |
| *BFP66_RS03515* | -2.49 | 1.49E-14 | hypothetical protein; |
| *BFP66_RS03825* | -2.50 | 3.35E-13 | glucuronate isomerase; |
| *BFP66_RS08865* | -2.51 | 0.000000358 | hypothetical protein; |
| *BFP66_RS02090* | -2.52 | 0.007653423 | ComF family protein; |
| *BFP66_RS04770* | -2.52 | 1.14E-67 | recombinase; |
| *BFP66_RS00080* | -2.53 | 4.96E-101 | rod shape-determining protein MreC; |
| *BFP66_RS04830* | -2.54 | 0.001701076 | inorganic pyrophosphatase; |
| *BFP66_RS05800* | -2.54 | 4.26E-12 | formate C-acetyltransferase/glycerol dehydratase family glycyl radical enzyme; |
| *BFP66_RS10215* | -2.55 | 3.89E-82 | NUDIX domain-containing protein; |
| *BFP66_RS04395* | -2.56 | 0.0000349 | IS110 family transposase; |
| *BFP66_RS10765* | -2.57 | 3.35E-14 | restriction endonuclease subunit M; |
| *BFP66_RS01070* | -2.60 | 4.79E-185 | colicin V production protein; |
| *BFP66_RS06060* | -2.60 | 2.82E-28 | rRNA pseudouridine synthase; |
| *BFP66_RS06870* | -2.61 | 1.14E-63 | DUF1694 domain-containing protein; |
| *BFP66_RS07110* | -2.62 | 0.0000822 | ArsR family transcriptional regulator; |
| *BFP66_RS02505* | -2.63 | 5.16E-09 | LLM class flavin-dependent oxidoreductase; |
| *BFP66_RS00175* | -2.74 | 1.06E-247 | hypothetical protein; |
| *BFP66_RS07800* | -2.74 | 0.009174461 | DUF910 domain-containing protein; |
| *BFP66_RS04300* | -2.75 | 2.22E-10 | tagatose-6-phosphate kinase; |
| *BFP66_RS02095* | -2.76 | 0 | ribosomal subunit interface protein; |
| *BFP66_RS08670* | -2.77 | 1.35E-41 | HIT family protein; |
| *BFP66_RS05220* | -2.77 | 1.57E-47 | DUF1149 domain-containing protein; |
| *BFP66_RS04330* | -2.79 | 2.7E-29 | protein lacX; |
| *BFP66_RS01465* | -2.81 | 1.16E-75 | nucleotide exchange factor GrpE; |
| *BFP66_RS06240* | -2.82 | 6.62E-52 | MarR family transcriptional regulator; |
| *BFP66_RS04810* | -2.84 | 5.75E-114 | LysR family transcriptional regulator; |
| *BFP66_RS03615* | -2.88 | 1E-149 | LacI family transcriptional regulator; |
| *BFP66_RS08025* | -2.88 | 9.16E-52 | transcriptional regulator NrdR; |
| *BFP66_RS07055* | -2.90 | 1.35E-43 | AraC family transcriptional regulator; |
| *BFP66_RS05975* | -2.95 | 3.82E-91 | 16S rRNA (cytidine(1402)-2'-O)-methyltransferase; |
| *BFP66_RS10675* | -3.02 | 0.00000158 | hypothetical protein; |
| *BFP66_RS07460* | -3.05 | 0.0000461 | DUF1294 domain-containing protein; |
| *BFP66_RS03000* | -3.05 | 5.7E-13 | carbohydrate ABC transporter permease; |
| *BFP66_RS07730* | -3.06 | 1.11E-21 | TetR/AcrR family transcriptional regulator; |
| *BFP66_RS09855* | -3.09 | 0.00000345 | PTS sugar transporter subunit IIB; |
| *BFP66_RS02710* | -3.11 | 1.99E-74 | UDP kinase; |
| *BFP66_RS05755* | -3.12 | 1.11E-200 | type 1 glycerol-3-phosphate oxidase GlpO; |
| *BFP66_RS08690* | -3.18 | 2.75E-28 | tRNA (adenosine(37)-N6)-threonylcarbamoyltransferase complex ATPase subunit type 1 TsaE; |
| *BFP66_RS07220* | -3.19 | 0.0000762 | PadR family transcriptional regulator; |
| *BFP66_RS08535* | -3.19 | 1.41E-16 | hypothetical protein; |
| *BFP66_RS10075* | -3.22 | 4.17E-28 | arginine repressor; |
| *BFP66_RS07870* | -3.26 | 2.21E-11 | membrane protein insertion efficiency factor YidD; |
| *BFP66_RS01810* | -3.29 | 1.57E-79 | multidrug DMT transporter; |
| *BFP66_RS01015* | -3.30 | 1.15E-185 | hypothetical protein; |
| *BFP66_RS00840* | -3.30 | 1.03E-262 | AraC family transcriptional regulator; |
| *BFP66_RS10430* | -3.31 | 0.00013016 | sigma-70 family RNA polymerase sigma factor; |
| *BFP66_RS00455* | -3.31 | 0.000131565 | sigma-70 family RNA polymerase sigma factor; |
| *BFP66_RS05920* | -3.42 | 2.03E-28 | DUF975 domain-containing protein; |
| *BFP66_RS04215* | -3.43 | 3.8E-98 | GTP pyrophosphokinase; |
| *BFP66_RS04960* | -3.44 | 0.00292823 | NisI/SpaI family lantibiotic immunity lipoprotein; |
| *BFP66_RS01065* | -3.46 | 7.04E-75 | hypothetical protein; |
| *BFP66_RS04270* | -3.46 | 5.2E-18 | site-specific integrase; |
| *BFP66_RS02210* | -3.46 | 2.09E-09 | PTS system mannose/fructose/N-acetylgalactosamine-transporter subunit IIB; |
| *BFP66_RS04315* | -3.47 | 1.18E-25 | PTS lactose/cellobiose transporter subunit IIA; |
| *BFP66_RS04765* | -3.48 | 1.75E-176 | recombinase; |
| *BFP66_RS01230* | -3.50 | 1.74E-75 | hypothetical protein; |
| *BFP66_RS08550* | -3.52 | 2.95E-83 | glyoxalase; |
| *BFP66_RS02990* | -3.53 | 1.74E-35 | LacI family transcriptional regulator; |
| *BFP66_RS03820* | -3.54 | 0.0000715 | 2-dehydro-3-deoxy-phosphogluconate aldolase; |
| *BFP66_RS10175* | -3.55 | 0.00000107 | thiol reductase thioredoxin; |
| *BFP66_RS10015* | -3.57 | 9.72E-214 | anaerobic ribonucleoside-triphosphate reductase activating protein; |
| *BFP66_RS09845* | -3.60 | 6.64E-15 | DUF3284 domain-containing protein; |
| *BFP66_RS08505* | -3.61 | 9.66E-253 | 6-phospho-beta-glucosidase; |
| *BFP66_RS01975* | -3.62 | 0 | Holliday junction resolvase RecU; |
| *BFP66_RS01545* | -3.62 | 2.03E-177 | transcriptional repressor; (Fur/Zur/PerR) |
| *BFP66_RS02620* | -3.64 | 1.05E-26 | hypothetical protein; |
| *BFP66_RS08545* | -3.64 | 0 | 6-phospho-beta-glucosidase; |
| *BFP66_RS04325* | -3.65 | 1E-202 | 6-phospho-beta-galactosidase; |
| *BFP66_RS04320* | -3.65 | 0 | PTS lactose transporter subunits IICB; |
| *BFP66_RS07305* | -3.66 | 5.98E-161 | PspC domain-containing protein; |
| *BFP66_RS04115* | -3.67 | 0 | class I SAM-dependent methyltransferase; |
| *BFP66_RS01960* | -3.68 | 5.12E-18 | cold-shock protein; |
| *BFP66_RS04255* | -3.69 | 8.62E-76 | XRE family transcriptional regulator; |
| *BFP66_RS09630* | -3.69 | 0 | NADPH-dependent FMN reductase; |
| *BFP66_RS04680* | -3.71 | 1.95E-147 | DNA-binding protein; |
| *BFP66_RS00445* | -3.74 | 0 | 50S ribosomal protein L17; |
| *BFP66_RS05490* | -3.74 | 0.00000356 | hypothetical protein; |
| *BFP66_RS01460* | -3.74 | 0 | heat-inducible transcriptional repressor HrcA; |
| *BFP66_RS08815* | -3.74 | 0 | acetolactate synthase, large subunit, biosynthetic type; |
| *BFP66_RS08705* | -3.77 | 5.08E-12 | IS110 family transposase; |
| *BFP66_RS10395* | -3.79 | 8.91E-11 | RNA-binding S4 domain-containing protein; |
| *BFP66_RS01280* | -3.85 | 4.62E-183 | site-specific integrase; |
| *BFP66_RS07860* | -3.85 | 3.12E-09 | tRNA (cytidine(34)-2'-O)-methyltransferase; |
| *BFP66_RS09930* | -3.87 | 0.003550207 | hypothetical protein; |
| *BFP66_RS07470* | -3.90 | 1.46E-166 | uridine kinase; |
| *BFP66_RS05750* | -3.95 | 3.06E-99 | aquaporin family protein GlpF; |
| *BFP66_RS09525* | -3.95 | 5.6E-50 | ribonuclease P protein component; |
| *BFP66_RS10760* | -3.95 | 0.000000159 | teicoplanin resistance protein VanZ; |
| *BFP66_RS01980* | -4.10 | 9.63E-11 | DUF1273 domain-containing protein; |
| *BFP66_RS06975* | -4.12 | 3.29E-93 | hypothetical protein; |
| *BFP66_RS08555* | -4.16 | 0 | 6-phospho-beta-glucosidase; |
| *BFP66_RS06825* | -4.16 | 0 | transcription antiterminator BglG; |
| *BFP66_RS06195* | -4.21 | 6.25E-265 | Crp/Fnr family transcriptional regulator; |
| *BFP66_RS05605* | -4.24 | 0.000411758 | DUF2969 domain-containing protein; |
| *BFP66_RS04800* | -4.25 | 0 | glucose-1-phosphate adenylyltransferase subunit GlgD; |
| *BFP66_RS07310* | -4.31 | 0 | hypothetical protein; |
| *BFP66_RS03455* | -4.38 | 6.82E-09 | hypothetical protein; |
| *BFP66_RS02775* | -4.42 | 0 | peptidase C69; |
| *BFP66_RS02470* | -4.49 | 6.66E-08 | hypothetical protein; |
| *BFP66_RS10735* | -4.52 | 0.0000157 | MarR family transcriptional regulator; |
| *BFP66_RS09850* | -4.61 | 0.009258005 | PTS lactose/cellobiose transporter subunit IIA; |
| *BFP66_RS03315* | -4.63 | 1.11E-190 | hypothetical protein; |
| *BFP66_RS03925* | -4.63 | 3.59E-20 | membrane protein; |
| *BFP66_RS02705* | -4.76 | 0 | endoribonuclease YbeY; |
| *BFP66_RS08580* | -4.78 | 1.7E-208 | Cof-type HAD-IIB family hydrolase; |
| *BFP66_RS01795* | -4.79 | 6.96E-149 | LacI family transcriptional regulator; |
| *BFP66_RS02625* | -4.83 | 9.79E-62 | TM2 domain-containing protein; |
| *BFP66_RS05830* | -4.89 | 0.00000174 | glycyl-radical enzyme activating protein; |
| *BFP66_RS10285* | -5.08 | 2.63E-37 | S4 domain-containing protein YaaA; |
| *BFP66_RS01745* | -5.10 | 9.56E-184 | universal stress protein; |
| *BFP66_RS02450* | -5.16 | 0.00051829 | DUF3270 domain-containing protein; |
| *BFP66_RS09585* | -5.17 | 3.01E-16 | LytTR family transcriptional regulator; |
| *BFP66_RS04310* | -5.21 | 1.95E-142 | transcription antiterminator lact; |
| *BFP66_RS04030* | -5.26 | 0 | transcriptional regulator Spx; |
| *BFP66_RS04760* | -5.27 | 0 | recombinase; |
| *BFP66_RS01000* | -5.34 | 3.91E-86 | Rrf2 family transcriptional regulator; |
| *BFP66_RS03970* | -5.44 | 0 | hypothetical protein; |
| *BFP66_RS09625* | -5.61 | 0 | NAD(P)H-dependent oxidoreductase; |
| *BFP66_RS05180* | -5.94 | 3.36E-73 | hypothetical protein; |
| *BFP66_RS08570* | -5.96 | 0 | PTS beta-glucoside transporter subunit EIIBCA; |
| *BFP66_RS07010* | -5.98 | 7.65E-48 | hypothetical protein; |
| *BFP66_RS02695* | -6.00 | 1.47E-29 | PTS glucose transporter subunit IIBC; |
| *BFP66_RS08575* | -6.49 | 0 | PRD domain-containing protein; |
| *BFP66_RS03630* | -6.67 | 0.0000039 | glutaredoxin-like protein NrdH; |
| *BFP66_RS02690* | -6.70 | 5.91E-21 | putative N-acetylmannosamine-6-phosphate 2-epimerase; |
| *BFP66_RS05315* | -6.88 | 3.05E-85 | polysaccharide deacetylase family protein; |
| *BFP66_RS02660* | -6.98 | 1.62E-41 | ferrous iron transporter A; |
| *BFP66_RS00910* | -7.00 | 5.88E-25 | PTS sugar transporter subunit IIC; |
| *BFP66_RS04335* | -7.06 | 5.97E-17 | hypothetical protein; |
| *BFP66_RS01375* | -7.13 | 0 | bifunctional acetaldehyde-CoA/alcohol dehydrogenase; |
| *BFP66_RS04805* | -7.36 | 0 | glucose-1-phosphate adenylyltransferase; |
| *BFP66_RS10715* | -7.38 | 0.001250086 | MerR family transcriptional regulator; |
| *BFP66_RS10185* | -7.70 | 1.89E-09 | hypothetical protein; |
| *BFP66_RS09860* | -7.74 | 8.23E-278 | transcription antiterminator BglG; |
| *BFP66_RS01805* | -7.99 | 0 | UDP-glucose--hexose-1-phosphate uridylyltransferase; |
| *BFP66_RS02185* | -8.05 | 0 | DUF402 domain-containing protein; |
| *BFP66_RS05570* | -8.39 | 0.004082177 | hypothetical protein; |
| *BFP66_RS02995* | -8.65 | 7.78E-51 | sugar ABC transporter permease; |
| *BFP66_RS01320* | -8.77 | 0.00015863 | ArsR family transcriptional regulator; |
| *BFP66_RS04650* | -8.83 | 5.12E-29 | hypothetical protein; |
| *BFP66_RS07030* | -8.84 | 4.61E-231 | sucrose phosphorylase; |
| *BFP66_RS05175* | -9.35 | 4.56E-152 | hypothetical protein; |
| *BFP66_RS02740* | -9.39 | 0.00000133 | preprotein translocase subunit SecG; |
| *BFP66_RS07035* | -9.50 | 7.81E-280 | carbohydrate ABC transporter permease; |
| *BFP66_RS00755* | -10.15 | 0.0000331 | hypothetical protein; |
| *BFP66_RS08510* | -10.66 | 0 | PTS cellobiose transporter subunit IIC; |
| *BFP66_RS07045* | -10.83 | 0 | sugar ABC transporter substrate-binding protein; |
| *BFP66_RS06170* | -10.90 | 2.36E-198 | YfcC family protein; |
| *BFP66_RS09330* | -11.55 | 0.000383163 | hypothetical protein; |
| *BFP66_RS02390* | -11.72 | 4.51E-11 | DUF1797 domain-containing protein; |
| *BFP66_RS01800* | -12.66 | 0 | galactokinase; |
| *BFP66_RS05785* | -12.96 | 2.23E-107 | glycerol dehydrogenase GldA; |
| *BFP66_RS02205* | -13.17 | 0 | beta-galactosidase; |
| *BFP66_RS10095* | -13.44 | 0.0000647 | hypothetical protein; |
| *BFP66_RS02975* | -13.59 | 7.01E-29 | DUF2500 domain-containing protein; |
| *BFP66_RS03575* | -14.07 | 0.000195594 | PTS N-acetylgalactosamine transporter subunit IIA; |
| *BFP66_RS04725* | -14.40 | 9.84E-11 | hypothetical protein; |
| *BFP66_RS07040* | -15.41 | 0 | sugar ABC transporter permease; |
| *BFP66_RS00450* | -19.75 | 0.00000224 | ISL3 family transposase; |
| *BFP66_RS04775* | -20.44 | 5.3E-29 | NUDIX hydrolase; |
| *BFP66_RS08520* | -24.75 | 1.18E-67 | PTS cellobiose transporter subunit IIA; |
| *BFP66_RS07050* | -30.05 | 0 | alpha-galactosidase; |
| *BFP66_RS06180* | -38.71 | 0 | ornithine carbamoyltransferase; |
| *BFP66_RS08515* | -40.76 | 0 | hypothetical protein; |
| *BFP66_RS06175* | -49.22 | 0 | carbamate kinase; |
| *BFP66_RS05790* | -51.82 | 1.86E-177 | glycerol dehydrogenase; |
| *BFP66_RS08530* | -53.00 | 6.62E-155 | PTS cellbiose transporter subunit IIC; |
| *BFP66_RS01370* | -55.30 | 0 | alcohol dehydrogenase AdhP; |
| *BFP66_RS06190* | -83.18 | 0 | arginine deiminase; |
| *BFP66_RS08525* | -88.84 | 0 | transcription antiterminator BglG; |
| *BFP66_RS06185* | -155.82 | 2.89E-226 | N-acetyltransferase; |
